# Leveraging T-cell receptor – epitope recognition models to disentangle unique and cross-reactive T-cell response to SARS-CoV-2 during COVID-19 progression/resolution

**DOI:** 10.3389/fimmu.2023.1130876

**Published:** 2023-05-31

**Authors:** Anna Postovskaya, Alexandra Vujkovic, Tessa de Block, Lida van Petersen, Maartje van Frankenhuijsen, Isabel Brosius, Emmanuel Bottieau, Christophe Van Dijck, Caroline Theunissen, Sabrina H. van Ierssel, Erika Vlieghe, Esther Bartholomeus, Kerry Mullan, Wim Adriaensen, Guido Vanham, Benson Ogunjimi, Kris Laukens, Koen Vercauteren, Pieter Meysman

**Affiliations:** ^1^ Adrem Data Lab, Department of Computer Science, University of Antwerp, Antwerp, Belgium; ^2^ Biomedical Informatics Research Network Antwerp (BIOMINA), University of Antwerp, Antwerp, Belgium; ^3^ Antwerp Unit for Data Analysis and Computation in Immunology and Sequencing (AUDACIS), University of Antwerp, Antwerp, Belgium; ^4^ Clinical Virology Unit, Department of Clinical Sciences, Institute of Tropical Medicine, Antwerp, Belgium; ^5^ Department of Clinical Sciences, Institute of Tropical Medicine, Antwerp, Belgium; ^6^ Centre for Health Economics Research & Modeling Infectious Diseases (CHERMID), Vaccine & Infectious Disease Institute (VAXINFECTIO), University of Antwerp, Antwerp, Belgium; ^7^ Department of General Internal Medicine, Infectious Diseases and Tropical Medicine, Antwerp University Hospital, Edegem, Belgium; ^8^ Global Health Institute, University of Antwerp, Antwerp, Belgium; ^9^ Department of Medical Genetics, University of Antwerp, Antwerp, Belgium; ^10^ Clinical Immunology Unit, Department of Clinical Sciences, Institute of Tropical Medicine, Antwerp, Belgium; ^11^ Antwerp Center for Translational Immunology and Virology (ACTIV), Vaccine and Infectious Disease Institute, University of Antwerp, Antwerp, Belgium; ^12^ Department of Paediatrics, Antwerp University Hospital, Antwerp, Belgium

**Keywords:** TCR repertoire analysis, CD8+ T-cell response, COVID-19, SARS-CoV-2 epitopes, immunoinformatics, cross-reactive T-cell response, machine learning models

## Abstract

Despite the general agreement on the significance of T cells during SARS-CoV-2 infection, the clinical impact of specific and cross-reactive T-cell responses remains uncertain. Understanding this aspect could provide insights for adjusting vaccines and maintaining robust long-term protection against continuously emerging variants. To characterize CD8+ T-cell response to SARS-CoV-2 epitopes unique to the virus (SC2-unique) or shared with other coronaviruses (CoV-common), we trained a large number of T-cell receptor (TCR) – epitope recognition models for MHC-I-presented SARS-CoV-2 epitopes from publicly available data. These models were then applied to longitudinal CD8+ TCR repertoires from critical and non-critical COVID-19 patients. In spite of comparable initial CoV-common TCR repertoire depth and CD8+ T-cell depletion, the temporal dynamics of SC2-unique TCRs differed depending on the disease severity. Specifically, while non-critical patients demonstrated a large and diverse SC2-unique TCR repertoire by the second week of the disease, critical patients did not. Furthermore, only non-critical patients exhibited redundancy in the CD8+ T-cell response to both groups of epitopes, SC2-unique and CoV-common. These findings indicate a valuable contribution of the SC2-unique CD8+ TCR repertoires. Therefore, a combination of specific and cross-reactive CD8+ T-cell responses may offer a stronger clinical advantage. Besides tracking the specific and cross-reactive SARS-CoV-2 CD8+ T cells in any TCR repertoire, our analytical framework can be expanded to more epitopes and assist in the assessment and monitoring of CD8+ T-cell response to other infections.

## 1 Introduction

The emergence of a severe acute respiratory syndrome coronavirus 2 (SARS-CoV-2) in 2019 has led to the most prominent pandemic in recent history. The SARS-CoV-2 infection manifests as coronavirus disease (COVID-19) with varying symptoms and severity and has caused substantial deaths all over the world.

The adaptive immune system is responsible for generating specific immunity against a viral infection. There is growing evidence that in case of SARS-CoV-2, T cells in particular might play a key role in infection control (1–3) even without seroconversion (4, 5), in moderation of COVID-19 severity (6, 7), and in the durability of natural (8, 9) and vaccination-induced (10, 11) immunity, including protection against viral variants. Previously, coronavirus-specific T-cell responses were already described as an important factor in the long-term immunity during SARS and MERS outbreaks (12–14), with some of the T cells exhibiting robust cross-reactivity against SARS-CoV-2 17 years later after the original infection (15). Pre-infection presence of CD4+ (16, 17) and CD8+ (18, 19) T cells that broadly recognize epitopes of SARS-CoV-2 due to relatedness with previously encountered viruses have been widely reported. Furthermore, both cross-reactive CD4+ (20, 21) and CD8+ (22, 23) T cells were suggested to have a protective effect in some individuals. Nevertheless, there are also conflicting findings questioning the clinical benefits of cross-reactive CD4+ (24–26) and CD8+ (27) T cells for other patients. Consequently, it is important to investigate the clinical impact of pre-existing SARS-CoV-2-specific T cells and to be able to distinguish them from *de novo* responding T cells. In particular, understanding the contribution of CD8+ T-cell clones with a particular specificity could guide the design of new generation vaccines or booster regimens to supplement weakening antibody-mediated neutralization (due to viral escape mutations) with an enhanced T-cell response and help stratify individuals into risk groups.

High-throughput T-cell receptor (TCR) sequencing paired with TCR-epitope mapping enables insights into an individual’s TCR repertoire composition. However, only a handful of TCR sequences, so-called “public” TCRs, are found across different individuals, while the majority of a TCR repertoire consists of “private”, unique to an individual, TCRs. Experimental assessment of epitope specificity of every single “private” TCR is not feasible due to the high inter- and intrapersonal diversity of TCRs. Accordingly, despite the ongoing efforts of sequencing studies to decipher “public” (28–30) and “private” (29–32) SARS-CoV-2 TCR sequences, specificity of most disease-associated T cells is yet to be resolved.

Recently, computational recognition models have been developed to connect T cells with their target epitopes (33–36). These models are based on the concept that TCRs recognizing the same epitope tend to have similar amino acid sequences (37). The advantage of the models is their ability to extract TCR-epitope interaction patterns from limited available experimentally validated data and to extrapolate them to previously unencountered TCRs. Thus, TCR repertoires can be easily screened to find potential epitope specificity of the unknown “private” and “public” TCRs. Here, we leverage such recognition models to track SARS-CoV-2 epitope-specificity in publicly available bulk TCR repertoires of COVID-19 patients (28) and in sorted CD4+ and CD8+ TCR repertoires of newly recruited COVID-19 patients. We report on the differential evolution of CD8+ T-cell response to unique SARS-CoV-2 epitopes (SC2-unique) and SARS-CoV-2 epitopes that are shared with other coronaviruses (CoV-common) in patients with critical and non-critical COVID-19 presentation.

## 2 Materials and methods

### 2.1 SARS-CoV-2 epitope-TCR TCRex recognition models

#### 2.1.1 Collection of the public TCR data with known SARS-CoV-2 epitope-specificity

A collection of experimentally validated TCR-epitope pairs was established by combining two primary sources: (1) The VDJdb database which contained tetramer-derived data from Shomuradova et al. (31); access date: May 26th, 2020]; (2) The ImmuneCODE collection from Adaptive Technologies and Microsoft which contained pairs derived through Multiplex Identification of Antigen-Specific T-Cell Receptors Assay (MIRA) (29); access date: June 25th, 2020].

For all extracted pairs, several data curation steps were performed. All pairs matching more than one possible SARS-CoV-2 epitope were removed from the training data. Only valid TCR sequences that could be matched to IMGT standard were kept. To meet an internal TCRex limit, 5000 unique TCRs were selected randomly for epitope HTTDPSFLGRY.

#### 2.1.2 TCRex recognition model training and application

The paired TCR-epitope dataset was used to train a set of TCRex models using the standard procedures as described in (36). In brief, for each epitope, a separate random forest model based on common physicochemical properties was trained using a positive training dataset, which consisted of TCRs experimentally validated to recognize this epitope (ref. to subsection 2.1.1). All constructed models satisfied the TCRex criteria for positive training data, which mandated the inclusion of a minimum of 30 distinct epitope-specific TCRs. The size of the positive training dataset varied from 36 to 5000 TCRs for different models (median=185, **Figure S1A**). For each model, a negative training dataset included 10 times more unique TCRs than a positive training dataset (with no overlapping TCRs) to resemble inherent underrepresentation of epitope-specific TCRs in full TCR repertoires and to maintain the same positive to negative data ratio, regardless of the number of available epitope-specific TCRs. Models were then evaluated using a 10-fold cross-validation, and only models with area under the receiver operating characteristic curve (AUC ROC) and average precision of at least 0.7 and 0.35, respectively, were retained, as suggested by TCRex quality criteria. Performance of all retained models was comparable regardless of the size of the training data (**Figure S1B**). This observation likely reflects a limited diversity of TCR sequences recognizing those epitopes since a recent study has demonstrated that a high degree of similarity in training TCR sequences rather than their number is strongly connected to the performance of models (38). All models used in this paper are available in the online TCRex tool (https://tcrex.biodatamining.be/), where any TCR repertoire data can be uploaded and one or more of 47 SARS-CoV-2 epitopes can be selected to get the predictions for those particular epitopes. With the web tool, it is also possible to train new prediction models for other epitopes of interest provided that the user has the appropriate training and test data.

TCRex models were then applied to the sequenced TCR repertoires of COVID-19 patients. Hits with a TCRex score greater than 0.9 and a baseline prediction rate (BPR) lower than 1e-4 were considered putative epitope-specific TCR sequences as per default TCRex filtering criteria. The BPR threshold represents the fraction of TCRs specific to a pathogen-derived epitope in a healthy TCR repertoire and thus controls for the number of false positive predictions, making the models applicable to TCR repertoires of both healthy individuals and patients. Since the paired TCR-epitope dataset used to train TCRex models consists entirely of major histocompatibility complex class I (MHC-I) restricted epitopes, valid predictions are only expected for CD8+ TCRs.

### 2.2 Patient data

#### 2.2.1 “Split” dataset: patient cohort and separate TCR repertoire sequencing of pre-sorted CD4+ and CD8+ T cells

The “split” dataset comprises separate CD4+ and CD8+ TCR repertoire sequences from patients with different COVID-19 severities collected during the initial stages of disease progression (the first 4 weeks after symptom onset). To this end, blood samples were collected from participants recruited in the IMSEQ study (NCT04368143), a prospective cohort study of COVID-19 patients admitted at the Antwerp University Hospital, Belgium (UZA) (the study was approved by the Institutional Review Board (IRB) of the Institute of Tropical Medicine (ITM) and UZA EC: number 20/12/135; ClinicalTrials.gov ID: NCT04368143). The inclusion criteria were: (1) having a laboratory-confirmed SARS-CoV-2 infection; (2) being older than 18; (3) providing written informed consent. COVID-19 severity was assigned based on the worst symptoms observed during the entire course of the disease.

For TCR sequencing of the COVID-19 patients, individuals exceeding the age of 65 and individuals diagnosed with or treated for oncologic conditions were excluded. We further selected patients that had donated at least two consecutive blood samples taken at least two days apart, the first of which within 16 days of symptom onset. As a result, 11 individuals with confirmed SARS-CoV-2 infections were retained including 7 patients classified as moderate, 1 as severe, and 3 as critical according to WHO grading (39) summarized in **Table S1**. Characteristics and sampling time points of retained study volunteers are summarized in **Table S2**.

At each time point, whole blood samples were obtained using three 9 mL S-Monovette^®^ lithium heparin tubes (Sarstedt). The PBMC fraction was isolated using LymphoprepTM (StemCell technologies), before cryopreserving aliquots in liquid nitrogen until further use.

After thawing, CD4+ and CD8+ T cells were positively selected using magnetic MicroBeads (Miltenyi Biotec), as described by the manufacturer. Counting was done manually on Trypan blue-stained cells using C-Chip counting chambers (NanoEnTek). All samples contained at least 200.000 viable cells and were stored in DNA/RNA shield (Zymo) at -80°C. Total RNA was extracted using Quick RNA microprep kit (Zymo) following the manufacturer’s protocol, eluted in 18µl DNAse/RNAse free H2O. The RNA concentrations were determined with Qubit RNA HS assay kit (Thermo Fisher Scientific). Each sample was split into triplicates (i.e., 3 vials of 5µl) that were used as library prep input. TCR library prep was done with QIAseq^®^ Immune Repertoire RNA Library and QIAseq^®^index kit (Qiagen, Venlo, Netherlands) that amplifies TCR alpha, beta, gamma, and delta chains. After quality control using TapeStation (Agilent, Santa Clara, CA, USA), concentration was measured with the Qubit dsDNA HS Assay kit (Thermo Fisher Scientific). For sequencing, each library was equivolume pooled. The pool was diluted to 4 nM and denatured. 1.1 pM of denatured library pool was run on the NextSeq 500 (NextSeq 500/550 Mid Output Kit v2.5, Illumina Netherlands) using 300 cycles with a pair-end 261-8-8-41 base read.

#### 2.2.2 “Mixed” dataset: collection of the publicly available bulk TCR repertoire sequences (CD4+ and CD8+ together)

A complementary “mixed” dataset (28), which contains TCR repertoires sequenced in bulk, without prior T-cell sorting into CD4+/CD8+, was downloaded from the iReceptor gateway (40) [access date: July 13th, 2020]. It features longitudinal (from week 1 to 8 after symptom onset) samples taken from patients with active disease and single time point samples from those that have recovered (week 4+ after symptom onset), summarized in **Table S2**. Patients classified as “severe” in the original study were reclassified as “critical” to match WHO grading (39) (**Table S1**). The data of asymptomatic individuals were excluded as their TCR response lies outside of the scope of our study. In total, TCR repertoires of 36 individuals were included (**Table S2**): 24 non-critical patients (16 recovered individuals who had mild COVID-19, 7 active patients with moderate COVID-19 and 1 patient with mild COVID-19 who had data points from both active and recovery phase of the disease) and 11 active patients with critical COVID-19 presentation. Noteworthy, critical disease resulted in the death of 5 out of 11 critical patients.

#### 2.2.3 Merged dataset: CD8+ and CD4+ TCR repertoires of the “split” dataset together with CD8+ TCR repertoires of the “mixed” dataset

Patient data analyzed in this study were assembled by combining TCR repertoires from internally produced “split” and publicly available “mixed” datasets into one merged dataset of 46 TCR repertoires of symptomatic COVID-19 patients. To make the number of data points per week more comparable between disease severity groups (**Figures S2A, B**), all individuals were divided into two groups (**Table 1**): critically ill (14 patients with critical COVID-19 severity) and non-critically ill (32 patients with mild, moderate, or severe COVID-19 severity). The median age of active critical and non-critical patients was 65 and 56.5, respectively. All available data points from the “split” dataset (11 symptomatic patients) were also used separately to verify whether the predictions of constructed recognition models are specific for the CD8+ T-cell subset.

**Table 1:** Summary of the datasets used for the analysis in this study.

Patients/Datasets		“mixed”	“split”	Merged
Critical	active	11	3	14
Non-critical	active	8*	8	16*
	recovered	17*	0	17*

*1 patient has data points from both stages, active and recovery.

To compensate for shifts in the response onset due to diverse times of admission and differences in the number of available data points for each patient, in further analysis of TCR repertoire metrics, only the maximum values of each week were considered when multiple time points were available for the same person during every time interval, (i.e., maximum TCR fraction between days 1-7 for week 1, maximum number of recognized epitopes between days 8-14 for week 2, etc.). Consequently, out of 32 individuals in the non-critical patient group, 15 patients had data points from the active stage of COVID-19, 16 – from the recovery stage, and 1 – from both stages (**Table 1**). All 14 critically ill patients remained sick throughout the entire duration of the study (up to 8 weeks) (**Table 1**).

### 2.3 TCR repertoire data processing and analysis

Demultiplexing of the sequencing data, unique molecular identifier (UMI) correction and generation of the UMI consensus for the “split” dataset were performed using MiNNN v.10.1 (https://minnn.readthedocs.io/). As three technical replicate experiments were conducted for each sample, only those TCR sequences that occurred in at least two out of three replicates were kept. Out of the selected replicates, the one with the highest total TCR count was retained for the downstream analysis.

Further steps were identical for both “split” and “mixed” datasets. TRB (T cell receptor beta gene) clonotype annotation was performed using MiXCR v.3.0.13 with the default input parameters (41). Only those TCRs that occurred at a frequency of at least 1 in 100 000 were retained, to compensate for the different sequencing depths between studies. Metadata was made uniform so that the time points are annotated by weeks after the onset of symptoms. All the data processing, comparisons and statistical analysis were performed using standard python3 libraries. Code necessary to enable the reproduction of the processing and analysis steps can be found in GitHub repository (https://github.com/apostovskaya/CovidTCRs/tree/main/src).

### 2.4 SARS-CoV-2 epitopes

To establish the “uniqueness” of each epitope in our database, we compared the presence of every SARS-CoV-2 epitope against protein sequences of 5 clinically relevant human coronaviruses (SARS-CoV, HCoV-229E, HCoV-HKU1, HCoV-NL63, HCoV-OC43) and 114 other *Nidovirales* species. Bat coronavirus RaTG13 (Ra4991), which is considered to be one of the closest relatives of SARS-CoV-2 due to 96.2% nucleotide sequence identity (42), was not present among compared species. These data were retrieved from the Corona OMA Orthology Database (43), where the used protein amino acid sequences for SARS-CoV-2 correspond to Genbank accession GCA_009858895.3, and the protein amino acid sequences for SARS-CoV to GCA_000864885.1. SARS-CoV-2 epitopes were matched to all proteins of all 119 species with an exact match, as the degree of variation allowed in the epitope space while retaining TCR recognition is still an unsolved question. Sequence identity between proteins was established using a pairwise protein BLAST. Matches across all species for each epitope were tallied, and the annotation for SARS-CoV-2 was retained: SARS-CoV-2 epitopes that occur only in 1 species (SARS-CoV-2) were labeled as “SARS-CoV-2-unique” (SC2-unique) and all others as “common for coronaviruses” (CoV-common). As SARS-CoV-2 genome evolves, some epitopes of the original variant might stop being SC2-unique in the later emerging variants. Since all the samples analyzed in the present study had been collected before August 2020, prior to the appearance of the first recognized variant (alpha/B.1.1.7) in autumn 2020, thus mitigating the problem of SARS-CoV-2 genetic diversity, only the original SARS-CoV-2 sequence was used to assign epitope uniqueness.

### 2.5 T-cell receptor (TCR) metrics

Different approaches can be used to analyze TCR repertoires. In our case, we were specifically interested in CD8+ T cells recognizing SARS-CoV-2 epitopes. Thus, four parameters were selected as the most informative: CD8+ TCR repertoire depth, CD8+ TCR repertoire breadth, CD8+ T-cell response diversity and CD8+ T-cell response redundancy. Repertoire depth was described as the relative frequency with which TCR sequences with a certain predicted specificity occur in the entire TCR repertoire. Repertoire breadth was calculated as the number of unique TCR sequences with a certain predicted specificity divided by the size of the unique TCR repertoire. Response diversity was represented as the number of putatively recognized SARS-CoV-2 epitopes. Average response redundancy was estimated as TCR/Epitope ratio: the number of unique SARS-CoV-2-specific TCRs to the number of recognized SARS-CoV-2 epitopes. Response metrics were calculated separately for SARS-CoV-2-unique (SC2-unique) and common for coronaviruses (CoV-common) epitopes. Repertoire metrics were rescaled so that proportions are consistent across datasets. Additionally, log2 fold change of a repertoire depth was monitored to evaluate the magnitude of temporal intrapersonal changes. Accordingly, for every patient for whom longitudinal data was available, TCR repertoire frequencies were shifted by 1 to enable subsequent calculation of fold changes between consecutive weeks and log2- transformation.

## 3 Results

### 3.1 SARS-CoV-2 epitope-TCR recognition models are robust and performant

To construct SARS-CoV-2 epitope-TCR recognition models, a collection of experimentally validated TCR-epitope pairs was established. To this end, data derived from T cells, the specificity of which was identified with peptide-MHC tetramers (31) was combined with ImmuneCODE TCR-epitope pairs derived from sorting of antigen-stimulated and activated CD8+ T cells using MIRA (multiplex identification of TCR antigen specificity) assay (29). After curation of the data and quality filtering of the models, 47 distinct epitope TCRex models were retained for SARS-CoV-2. An overview of all models and their performance can be found in **Table S3**.

**Figure 1:**
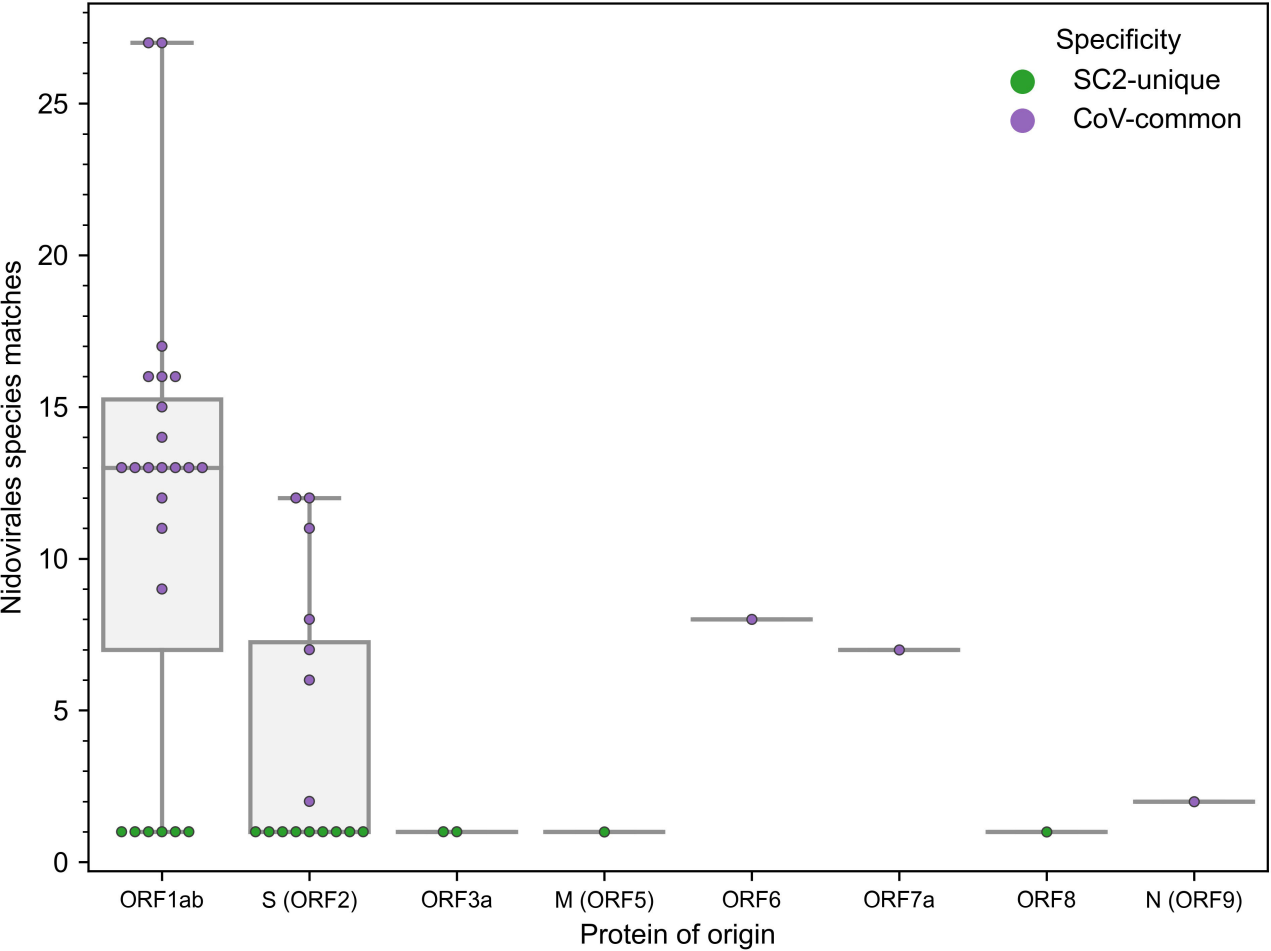
Distribution of the 47 epitopes for which TCR recognition models could be created across SARS-CoV-2 proteins (x-axis) and amount of exact amino acid epitope matches within the 119 species of the *Nidovirales* order (y-axis). TCR models for epitopes unique to SARS-CoV-2 (n=19) correspond to y=1.

The number of newly constructed TCRex models for SARS-CoV-2 epitopes almost equals 49 previously available TCRex models for all non-SARS-CoV-2 epitopes combined (36), indicating the vast amount of data that has been generated since the start of the pandemic compared to what has been collected for all prior pathogens and diseases. Twenty-four of these 47 epitopes match the SARS-CoV-2 replicase protein coded by ORF1ab, 16 match the SARS-CoV-2 spike protein encoded by ORF2 and the final 7 are distributed across the remaining proteins (**Figure 1**). In addition, 19 of the 47 epitopes are 100% unique to SARS-CoV-2 in our dataset of 119 *Nidovirales* species. As can be seen in **Figure 1**, the unique SARS-CoV-2 epitopes are not evenly distributed across the proteins. While only 6 out of 24 epitopes originating from the ORF1ab replicase protein are unique to SARS-CoV-2, there are 9 out of 16 epitopes derived from the spike protein that are specific to the virus. Although mutations are 5 times more frequent in the spike protein compared to the genomic average (44) and thus might weaken B-cell response, T-cell recognition appears to be retained against different variants including Omicron (11, 45, 46). Additionally, as previously evaluated for 3 SARS-CoV-2 variants, only 1 mutation overlapped with 52 MHC-I epitopes recognized by convalescent individuals (47). Together, this bodes well for the constructed TCRex models to stay relevant for the continuously emerging variants.

As we were integrating models from different resources and experimental methods, we wished to confirm whether models could extrapolate from the patterns in one data set to the other. Interestingly, one epitope had both tetramer (315 TCRs) and MIRA data (366 TCRs), namely YLQPRTFLL (YLQ). However, since TCRs in the experimental MIRA data were assigned to a group of 3 epitopes (YLQPRTFL, YLQPRTFLL, and YYVGYLQPRTF) and not just to YLQ, these 366 TCRs were excluded from the training data. Therefore, it was possible to evaluate the performance of the YLQ model (trained on the tetramer YLQ dataset) on an independent MIRA YLQ dataset containing experimentally validated TCR sequences that were not present in the training tetramer YLQ data. Out of 366 TCRs in the YLQ MIRA dataset, TCRex predicted 81 TCRs to be specific only to YLQ epitope and not to any other epitope present in TCRex (including both the 46 non-YLQ SARS-CoV-2 models and the 49 non-SARS-CoV-2 models). Out of these 81 TCRs, 46 TCRs (CDR3 and V/J genes) and 35 V/J combinations were not present in the training tetramer YLQ dataset. Moreover, some of the 366 TCRs may be associated with 2 other epitopes rather than with YLQ. Thus, (1) TCRex was able to correctly identify new, relative to the training data, TCRs; (2) a good fraction of YLQ-specific TCRs was detected; (3) the models are applicable to the TCR data of diverse origin.

### 3.2 Recognition models can be used to track epitope-specificity in CD8+ TCR repertoires

**Figure 2:**
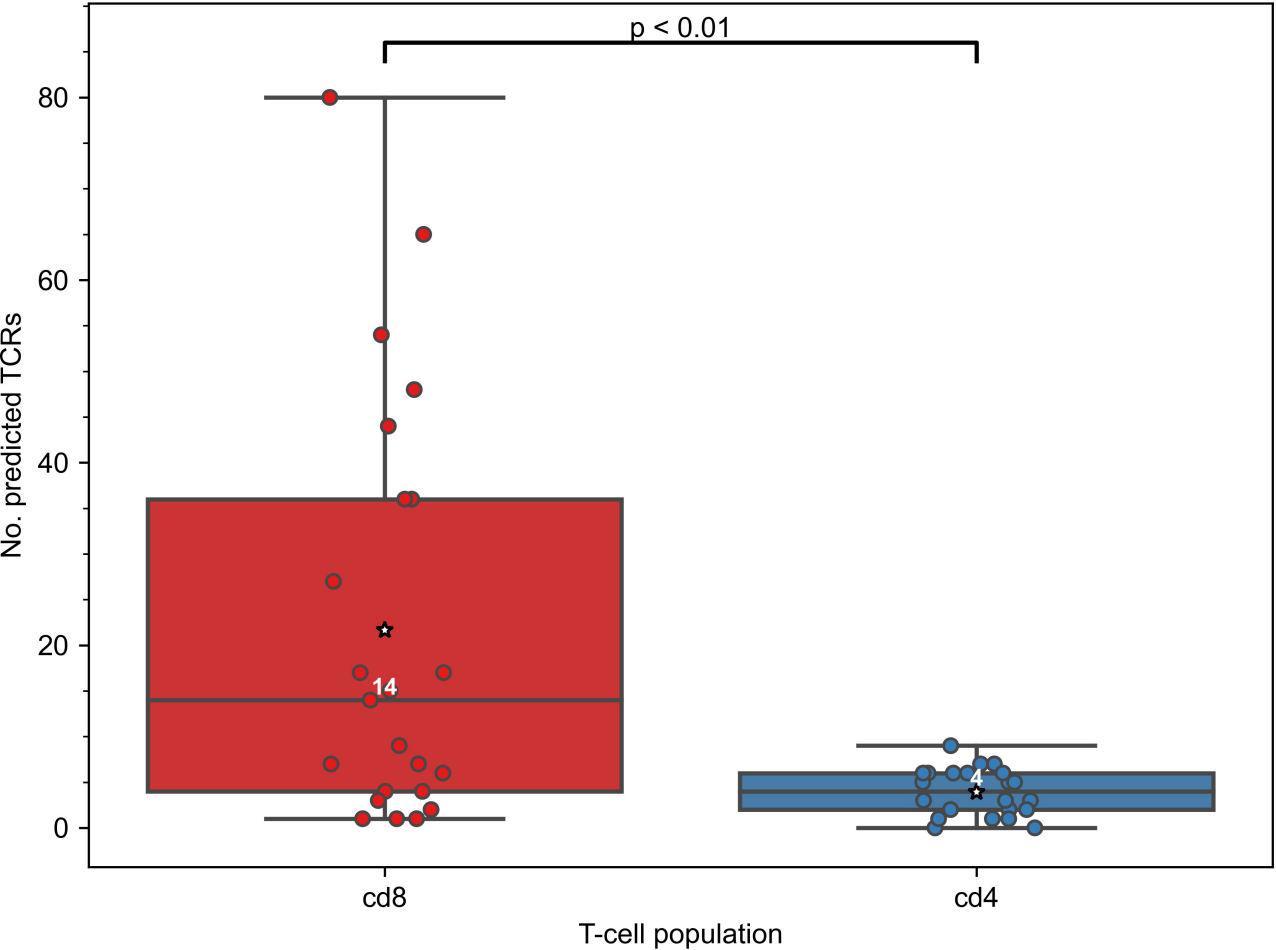
Constructed TCRex recognition models are suitable for the prediction of CD8+ T-cell specificity. As the models were built for epitopes presented in MHC-I, the number of TCRs predicted to recognize SARS-CoV-2 epitopes was significantly higher in CD8+ than in CD4+ T-cell repertoires when SARS-CoV-2 TCRex models were applied to an in-house COVID-19 patient “split” TCR dataset (Mann–Whitney U test p=0.001, nCD8 = 23, nCD4 = 22). White numbers specify the median number of the TCRs in a repertoire that were predicted by TCRex to be specific to SARS-CoV-2 epitopes; mean values are represented by a star.

As the constructed models predict specificity to epitopes presented on MHC-I molecules, we expected that TCRs predicted to recognize those epitopes will be enriched in CD8+ T cells. To validate this assumption, we generated CD4+ and CD8+ TCR repertoires for 11 COVID-19 patients (“split” dataset) at multiple time points and applied our 47 SARS-CoV-2 TCRex models to this data. As can be seen in **Figure 2**, the number of predicted SARS-CoV-2 reactive TCRs was indeed significantly higher in the CD8+ compared to the CD4+ T-cell population (Mann–Whitney U test p=0.001). There is, however, a small number of hits within the CD4+ population which is not unforeseen given some inefficiency inherent to magnetic cell sorting and common CDR3 sequences occurring in both the CD4+ and CD8+ populations (37, 48). The predominant signal in the CD8+ T cells confirms that the models are specific towards this subpopulation and thus suitable to track particularly the CD8+ T-cell response in any individual TCR repertoire, sequenced in bulk or after prior sorting.

### 3.3 Initial CD8+ T-cell response is similar in all patients, regardless of COVID-19 severity

In this study, we employed TCRex models to analyze COVID-19 TCR repertoires of 14 critically and 32 non-critically ill symptomatic patients. Prediction of putative SARS-CoV-2-specific CD8+ T cells identified 755 TCRs in the dataset cohort. Of these, 149 and 606 TCRs were found in samples from patients with critical and non-critical COVID-19 presentation, respectively.

Since the level of pre-existing T cells cross-reactive to SARS-CoV-2 and the swift mounting of T-cell responses had been postulated to influence COVID-19 progression (7, 49), we first assessed the initial size of SARS-CoV-2 specific CD8+ TCR repertoires. Therefore, the differences in the abundance of T-cell clones putatively recognizing MHC-I presented SARS-CoV-2 epitopes that are either unique to the virus (SC2-unique) or also occur in other *Nidovirales* species (CoV-common) were compared in patients with critical and non-critical symptomatic COVID-19. The prevalence of CD8+ TCRs with a certain specificity was described as relative frequencies with which those TCRs occur in the collected TCR repertoires, i.e., the depth of the responding TCR repertoire.

**Figure 3:**
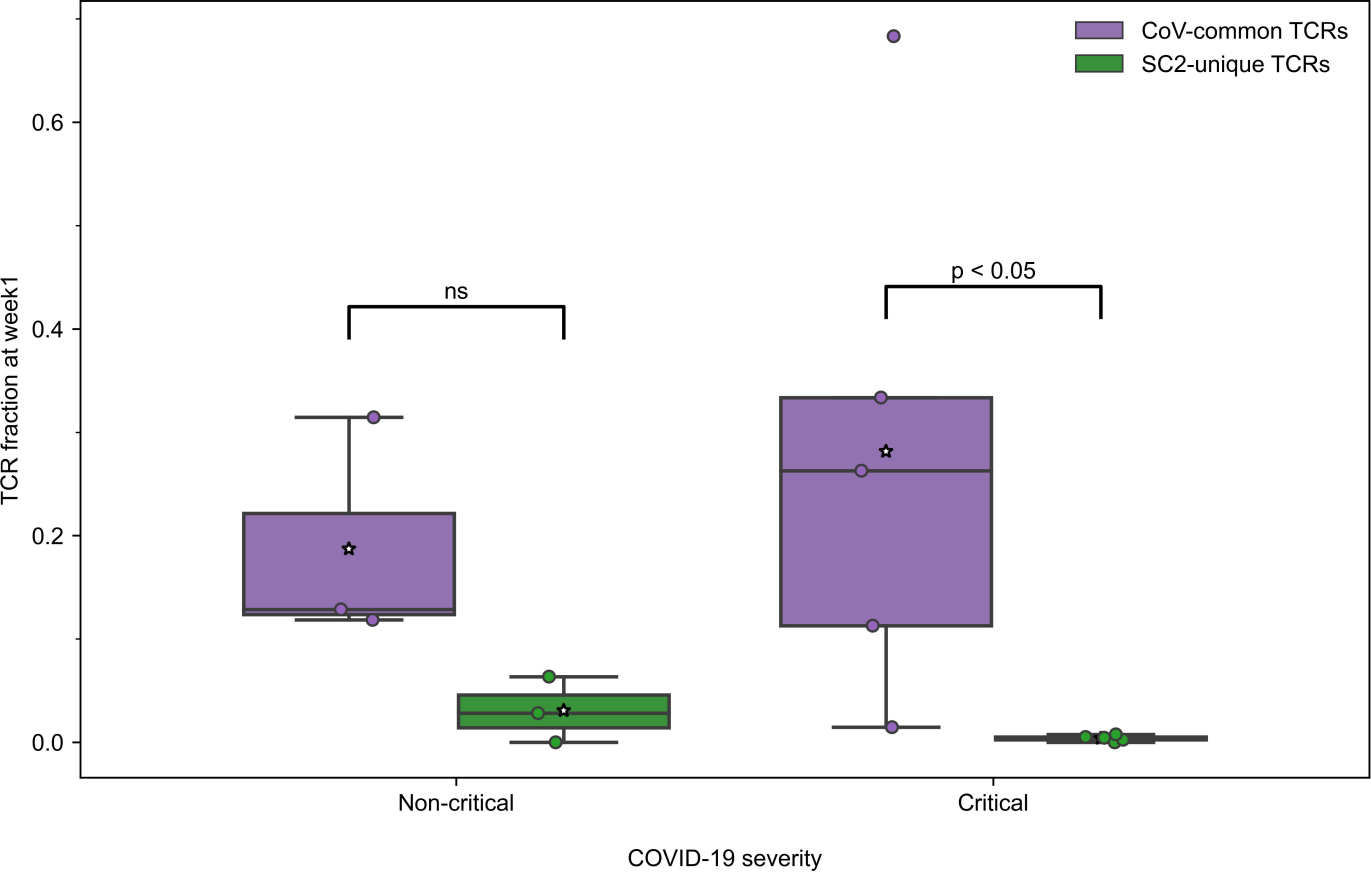
During the first week of COVID-19, relative frequencies of TCRs (depth of the repertoire) predicted to recognize CoV-common epitopes were higher than of TCRs putatively specific to SC2-unique epitopes in all patients with non-critical (Bonferroni corrected Mann–Whitney U test p=0.16, n=3) and critical (Bonferroni corrected Mann–Whitney U test p=0.048, n=5) COVID-19. Mean values are represented by a white star.

All active patients, regardless of the disease severity, had more TCRs specific to CoV-common than SC2-unique epitopes only during week 1 after the symptom onset (**Figure 3**) and not at any other subsequent week of the disease (**Table S4**). This disparity was more pronounced in critical patients (n=5) for whom the difference was statistically significant (Bonferroni corrected Mann–Whitney U test p=0.048, AUC=1, **Figure 3**). In the non-critical group (n=3), the frequency of putative SC2-unique TCRs was already higher than in the critical group, although not significantly (**Table S5**). In addition, no significant difference in the frequency of CoV-common TCRs, the total number of TCRs and the percent of unique TCRs was detected between critical and non-critical groups at this time (**Table S5**). We have also observed a matching predominance of CoV-common over SC2-unique TCRs within critical and non-critical patient groups at week 1 in a publicly available single-cell TCR dataset (50) (**Figure S9**). This larger independent dataset (**Supplementary material 1**), which comprised 20 critical and 30 non-critical patients, allowed us to increase the confidence in the discovered disparity in the abundance of SC2-unique and CoV-common TCRs. Together, these findings suggest that prior to the encounter with SARS-CoV-2, many individuals already have a substantial TCR repertoire dedicated to CoV-common epitopes, which is the first to respond to SARS-CoV-2.

### 3.4 Putative SARS-CoV-2 specific CD8+ T cells are mounted within the first two weeks of COVID-19 only in non-critical patients

To investigate whether the differences between frequencies of SC2-unique and CoV-common TCRs ceased after week 1 due to an increase of SC2-unique or decrease of CoV-common TCR repertoire, we studied changes in the depth (frequency of specific TCRs) and breadth (percent of specific TCRs out of unique TCRs) of respective TCR repertoires within each patient group. We observed that critical and non-critical patients had the opposite dynamics during the first two weeks (summarized in **Table 2**): the median breadth and depth of SC2-unique TCR repertoires increased from week 1 to week 2 only in non-critical patients (**Figures S3A, C**), and the median breadth and depth of the CoV-common TCR repertoire decreased from week 1 to week 2 only in critical patients (**Figures S3B, D**). Furthermore, we compared intragroup changes in the diversity of the response – the number of different SC2-unique and CoV-common epitopes being recognized by an individual TCR repertoire. We reasoned that an increase in those parameters could be an indirect indication of the *de novo* activation of T cells as opposed to the expansion of already activated T-cell clones. We discovered that non-critical patients, unlike critical ones, generally recognize 2 times more CoV-common and 3 times more SC2-unique epitopes at week 2 compared to week 1 (**Table 2**; **Figures S3E, F**). Additionally, only the redundancy of SC2-unique response increased 10 times between weeks 1 and 2 in non-critical patients alone (**Table 2**; **Figures S3G, H**).

**Table 2:** Changes in the SARS-CoV-2 TCR repertoire between week 1 and week 2.

Specificity	Parameter		Patients	
			Critical	Non-critical
CoV-common	TCR repertoire	depth (TCR frequency)	↓	=
		breadth (% of specific TCRs)	↓	=
	Response	diversity (No. recognized epitopes)	↓	↑
		redundancy (No. unique TCRs per 1 epitope)	=	=
SC2-unique	TCR repertoire	depth (TCR frequency)	= (0)	↑
		breadth (% of specific TCRs)	↓	↑
	Response	diversity (No. recognized epitopes)	↓	↑
		redundancy (No. unique TCRs per 1 epitope)	=	↑

All these results consistently point out that even though the CD8+ T-cell response in all symptomatic patients, regardless of the disease severity, starts with the mounting of (pre-existing) T cells specific to CoV-common epitopes during the first week after the symptom onset, only individuals with non-critical COVID-19 appear to be effectively activating and expanding T cells recognizing SC2-unique epitopes during the first two weeks of the disease. Intriguingly, we did not observe the dominance of T cells specific to CoV-common epitopes. CoV-common TCR repertoire depth and breadth and CoV-common response diversity in non-critical patients did not increase, despite the growth in the number of recognized epitopes. This could be attributed to two continuous opposing processes: depletion of already activated (pre-existing) CoV-common T cells and *de novo* activation of T cells recognizing previously unseen CoV-common epitopes.

### 3.5 COVID-19 severity is moderated by SC2-unique TCR repertoire depth, redundancy of SC2-unique and diversity of CoV-common TCR responses

**Figure 4:**
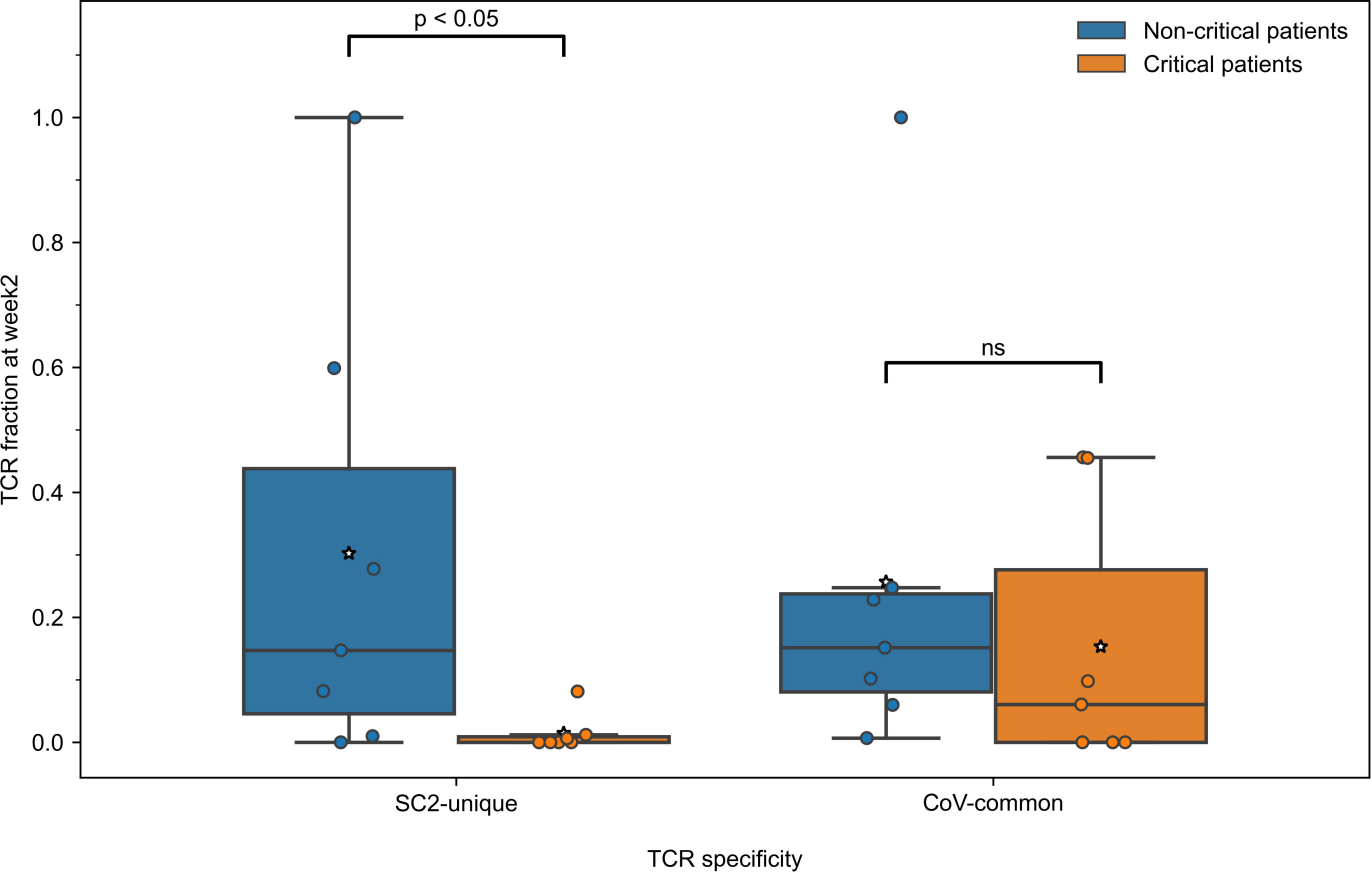
During the second week of COVID-19, relative frequencies of TCRs (depth of the repertoire) predicted to recognize SC2-unique [Bonferroni corrected Mann–Whitney U test p=0.039, n=14 (7 critical and 7 non-critical)] but not CoV-common (Bonferroni corrected Mann–Whitney U test p=0.456, n=14 [7 critical and 7 non-critical)] epitopes were significantly higher in symptomatic non-critical patients compared to critically ill patients. Mean values are represented by a white star.

Since the development of CD8+ T-cell response to SARS-CoV-2 differed during the first two weeks depending on the disease severity, we further compared the response levels between 7 critically and 7 non-critically ill patients at week 2, when TCRs to both previously seen and newly encountered epitopes are expected to have been activated and expanded. Pairwise comparisons between all the individuals from non-critical and critical groups revealed that in 81.6% of pairs, non-critical patients had significantly higher frequencies of TCRs specific to SC2-unique (Bonferroni corrected Mann–Whitney U test p=0.039, **Figure 4**) but not CoV-common (Bonferroni corrected Mann–Whitney U test p=0.456, **Figure 4**) epitopes than critically ill patients during week 2 of COVID-19. Moreover, we observed a significant difference in the total number of TCRs between two patient at week 2 (Bonferroni corrected Mann–Whitney U test p=0.033, AUC=0.878, **Figure S4A**), while no such difference was observed at week 1 (**Table S5**). Importantly, the proportion of unique TCRs in the repertoires remained consistent across the groups (**Figure S4B**). When we examined non-critical and critical patients (n=4) from the independent single-cell dataset (50), who had higher prevalence of SC2-unique TCRs (**Supplementary material 1**), we confirmed that only non-critical TCR repertoires underwent clonal expansion to a large clone size, while singleton TCRs constituted the bulk of critical patients’ TCR repertoires (**Figure S10A**). SC2-unique T cells of both critical and non-critical patients (n=2) generally expressed *GNLY*, *PFR1* and *GZMB* indicative of an effector phenotype (**Figure S10B**). T cells with CoV-common TCRs had a mix of effector, memory and naive phenotypes with predominance of naive and effector T cells in critical and non-critical patients, respectively (**Figure S10B**).

**Figure 5:**
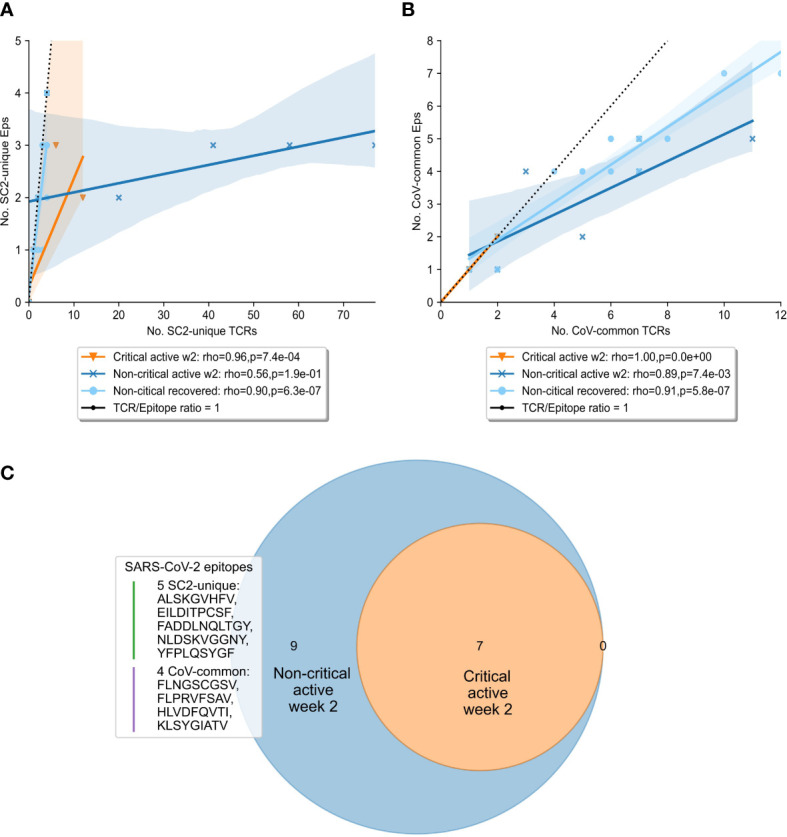
Diversity and redundancy of the response differed between patients with critical and non-critical symptomatic COVID-19 during the second week of the disease. **(A, B)** Redundancy (the number of unique TCRs recognizing the same epitopes) was significantly higher in non-critical (dark blue) compared to critical (orange) patients and disappeared once patients recovered (light blue). This was true for both **(A)** SC2-unique and **(B)** CoV-common epitopes. **(C)** 9 epitopes, out of which 5 are unique to SARS-CoV-2 (SC2-unique) and 4 are shared with other species of the *Nidovirales* order (CoV-common), were recognized exclusively by non-critical patients during the second week of COVID-19. No epitopes were recognized only by critical patients.

To gain further insights into the drivers behind the expansion of SARS-CoV-2 specific TCRs, we compared the diversity of the overall SARS-CoV-2 T-cell response in the symptomatic patients. Particularly, we sought to determine whether TCRs were specific to a limited pool of SARS-CoV-2 epitopes, or the number of targeted epitopes was increasing. At week 2, the number of recognized CoV-common but not SC2-unique epitopes was significantly higher in non-critical patients compared to critical patients (Bonferroni corrected Mann–Whitney U test p=0.026, AUC=0.796, **Table S5**). When this parameter was normalized for the repertoire size, there was no difference (**Table S5**) suggesting that the CD8+ T-cell response of critical patients was limited by the low number of available unique TCRs (diminished TCR diversity). Furthermore, non-critical patients, unlike critical patients (**Figures 5A, B**, median=1 for both epitope groups), exhibited redundancy in the T-cell response to both SC2-unique (**Figure 5A**, median=10 TCRs “per average epitope”, range=[1-26]) and, although much less pronounced, CoV-common (**Figure 5B**, median=2 TCRs “per average epitope”, range=[1-3]) epitopes. Later (week 3+), the redundancy of the response to both groups of epitopes was decreasing in active non-critical patients (**Figure S5**). This decrease could potentially indicate the ongoing resolution of the infection, as the redundancy completely disappeared once the patients had recovered (**Figures 5A, B**). During these later weeks, active critical patients also began to develop redundancy in the SC2-unique response (**Figure S5**), hinting that the lack of a timely SC2-unique T-cell response was not due to the absence of SC2-unique TCRs in their TCR repertoires.

Given that both patient groups had TCRs putatively targeting both SC2-unique and CoV-common epitopes, we set out to explore whether a specific set of recognized epitopes could be connected to milder COVID-19 cases. Unsurprisingly, no epitopes were found to be recognized by all patients within either disease severity group. As epitope recognition depends not only on TCR sequences but also on human leukocyte antigen (HLA) types present in an individual, it is highly probable that sets of recognized epitopes are unique to each patient. Nevertheless, 9 epitopes (5 SC2-unique and 4 CoV-common) were recognized exclusively by non-critical patients, albeit each epitope by only 1 or 2 individuals (**Figure 5C**). Those epitopes were recognized throughout the entire duration of the disease, by active or recovered non-critical patients.

Collectively, our results do not seem to indicate that pre-existing SARS-CoV-2 cross-reactive CD8+ T cells alone are associated with milder COVID-19 cases. Critically ill patients struggle to generate SC2-unique CD8+ TCRs and sustain CoV-common CD8+ TCRs. Conversely, CD8+ T-cells putatively recognizing SC2-unique epitopes are activated and expanded in non-critical patients by the second week of COVID-19. Finally, a diverse and redundant CD8+ T-cell response appears to be associated with less severe COVID-19 cases.

### 3.6 TCR diversity potential is reduced during COVID-19

**Figure 6:**
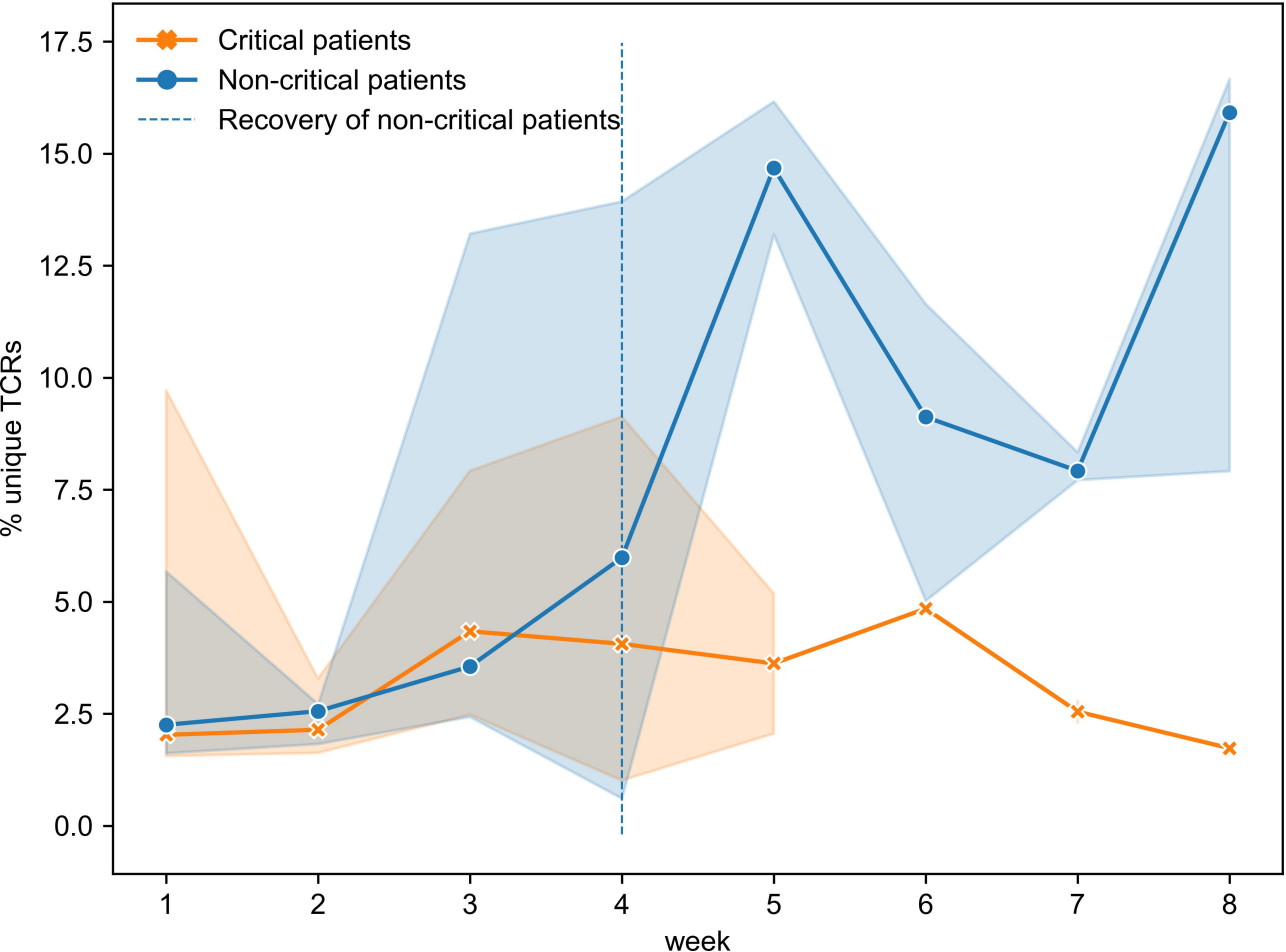
Proportion of unique TCRs was increasing significantly only in symptomatic non-critically ill patients (dark blue, Spearman rho=0.62, p=3e-05) and became significantly higher once patients started recovering (Mann–Whitney U test p=2e-04). Multiple inter- and intra-individual values combined within each disease severity group (critical: n=14, non-critical: n=32) are represented as tendency lines with a 95% confidence interval (shadow areas) when multiple data points were available at overlapping time points. The vertical dashed line (black) separates the active and recovery stages of the disease in non-critical patients.

To gain a better understanding of the SARS-CoV-2 associated changes in the overall TCR repertoire, we next analyzed longitudinal data spanning 8 weeks. As reported above, critical and non-critical symptomatic patients had comparable total numbers of sampled TCRs and proportions of unique TCRs at the beginning of their COVID-19. However, examination of all available data revealed that the proportion of all unique TCRs (irrespective of their epitope specificity) significantly increased in individuals with the non-critical disease over the entire period of the study (**Figure 6**, Spearman rho=0.62, p=3e-05) and became significantly higher after week 4, once patients entered recovery stage (Mann–Whitney U test p=2e-04, AUC=0.83). In contrast, TCR repertoires of critical patients, despite some trend for increase between weeks 3 and 6, on average remained less diverse even at week 8 of the disease (**Figure 6**, rho=0.08, p=0.69) indirectly supporting a previously reported correlation between disease severity and lymphopenia derived from a different type of analysis (51). These findings suggest that the number of CD8+ T cells is similarly reduced in both patient groups during the early phase of SARS-CoV-2 infection. This reduction persists in active critical patients but is restored in recovered non-critical patients.

### 3.7 Development of SARS-CoV-2 reactive CD8+ T-cell immunity in critical patients is dominated by TCRs predicted to target epitopes unique to SARS-CoV-2

To further investigate the most prominent dynamics of SARS-CoV-2 TCR repertoires, we disentangled longitudinal changes in SC2-unique and CoV-common TCRs in our patient cohorts with different COVID-19 severity. First, we considered only patients of whom samples were available from at least two different weeks (6 non-critical, 9 critical) to understand individual SARS-CoV-2 specific TCR repertoire evolution. For this subset, log2 fold change of TCR frequencies between consecutive data points was calculated (**Figures S6A, B**). This analysis revealed that the majority of non-critical (5/6) and critical (7/9) patients experienced decline in the frequency of CoV-common TCRs at least once during their active COVID-19. 33% (2/6) of non-critical and 56% (5/9) of critical patients didn’t have any changes in the depth of SC2-unique TCR repertoires. In all individuals, SC2-unique and CoV-common TCR repertoires were changing in the opposite directions 9 times, 5 times in the same direction and 8 times only one of the repertoires was changing between consecutive weeks while the other remained the same highlighting that TCR repertoires are constantly evolving. Noteworthy, the range of the magnitude of the change was comparable between SC2-unique and CoV-common TCR frequencies and between critical and non-critical groups.

**Figure 7:**
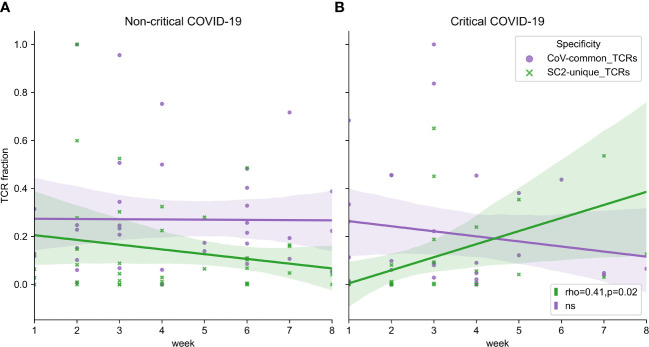
**(A)** Symptomatic non-critical patients demonstrated significant increases of neither SC2-unique (green, Spearman rho=-0.06, p=0.71) nor CoV-common (purple, Spearman rho=0.06, p=0.72) TCR repertoires when disease and recovery stages were evaluated together. **(B)** In contrast, relative frequencies (depth of the repertoire) of SC2-unique TCRs (green, Spearman rho=0.41, p=0.02) but not CoV-common TCRs (purple, Spearman rho=-0.10, p=0.58) were increasing significantly during disease progression in critical patients. Lines represent an estimate of the central tendency of multiple inter- and intra-individual values combined within each disease severity group (critical: n=14, non-critical: n=32) with a 95% confidence interval (shadow areas) in case multiple data points were available at overlapping time points.

Facing the limitation of our dataset wherein only 1 or 2 data points had been collected for most patients (**Figure S2C**), we attempted to extrapolate general group trends from the obtained longitudinal and single-point observations. Despite intra- and interpersonal variability of SARS-CoV-2 TCR repertoire expansion and contraction (**Figure S7**), there seemed to be some overall trends. In particular, multiple patients from both disease severity groups had a rise of SC2-unique and CoV-common TCR frequencies around weeks 2-3 (**Figures S8A, B**). The depth of CoV-common TCR repertoires also seemed to increase around week 6 in some individuals with critical and non-critical COVID-19 severity (**Figures S8A, B**). In the critical group, SC2-unique TCRs showed a tendency to increase in their frequency throughout the entire duration of the study despite sustained T-cell depletion (**Figure 7B**, Spearman rho=0.41, p=0.02). This was not the case for the non-critical patient group (**Figure 7A**, Spearman rho=-0.06, p=0.71) where the maximum frequency of SC2-unique TCRs was reached by weeks 2-3 (**Figure S8A**).

The most prominent breadth changes occurred exclusively in SC2-unique TCR repertoires, while the breadth of TCR repertoires reactive to CoV-common epitopes remained relatively stable in both patient groups during the entire study period (**Figures S8C, D**). Notably, the tendency for the increased breadth of SC2-unique TCRs was dominated by non-critical patients and mostly occurred within the first two weeks after symptom onset (**Figure S8C**). This trend was delayed in the critical group and was supported by only few individuals (**Figure S8D**).

Those findings reinforce that it is the build-up of the SARS-CoV-2 TCR repertoires, which is happening during the first two weeks of the disease, that might be crucial for differentiating COVID-19 severity. The SARS-CoV-2 specific CD8+ TCRs in critical patients seem to expand slower than in non-critical patients.

## 4 Discussion

Notwithstanding the general agreement on the importance of T cells during SARS-CoV-2 infection, the contribution of SARS-CoV-2-unique and cross-reactive T-cell responses towards modulation of COVID-19 severity remains not fully resolved. In this study, we combined our newly generated TCR sequences from COVID-19 patients hospitalized in a single center in Belgium with public datasets to gain insights into the specificity and evolution of CD8+ TCR repertoires in critical and non-critical symptomatic COVID-19 patients.

We observed that CD8+ T cells predicted to target CoV-common epitopes are mounted, despite T-cell depletion, in both critical and non-critical patients during the first week after symptom onset. Since the frequency of SC2-unique TCRs was significantly lower in our study samples at that time, we deduced that the depth of CoV-common TCR repertoires was higher in the first week due to more rapid clonal expansion of pre-existing cross-reactive memory CD8+ T cells. Therefore, individuals with previous exposure to coronaviruses may have formed memory CD8+ T cells that can rapidly respond to CoV-common epitopes from SARS-CoV-2, while *de novo* induced SC2-unique CD8+ T-cell immunity has not been developed yet. This explanation falls in line with previous reports where T cells recognizing SARS-CoV and seasonal coronaviruses were found to be cross-reactive to SARS-CoV-2 (16–19). Alternatively, individuals may have had previously developed cross-reactive CoV-common CD4+ T cells that might have facilitated more rapid CD8+ T cell development at the beginning of the SARS-CoV-2 infection, as has been recently demonstrated to be the case for antibody response to vaccination (52).

By the second week after symptom onset, activation and expansion of SC2-unique TCRs seem to have occurred in non-critical patients despite T-cell depletion. This conclusion can be made since at week 2 but not week 1 we observed (1) a significantly higher frequency of these TCRs in critical compared to non-critical patients; (2) equivalent depth of SC2-unique and CoV-common repertoires and (3) increased median number of recognized SARS-CoV-2 epitopes in non-critical patients. In contrast, critically ill patients experienced reduction in the breadth of CoV-common and SC2-unique TCR repertoires and respective response diversities. This disparity can partially be explained by lymphopenia which is known to be more pronounced in critical patients (51) and seems to affect CD8+ T-cell population more (6, 53). Previous research has provided evidence that T cells are dying during severe COVID-19 due to apoptosis (54), but the specificity of those T cells has not been extensively addressed. We further speculate that during lymphopenia in critical patients, SARS-CoV-2 recognizing rather than any CD8+ TCRs might be specifically depleted. If random TCRs were dying, the breadth of SARS-CoV-2 TCR repertoires would not have decreased, as specific TCRs constitute the minority of all unique TCRs in the repertoire. Markedly, recent study by Lee at al. (55) described a metabolically dominant cluster of CD8+ T cells which expressed markers suggestive of antigen-induced T cell apoptosis. According to grouping of lymphocyte interactions by paratope hotspots (GLIPH) analysis, TCRs in this cluster were specific to SARS-CoV-2. When we compared the epitope recognized by these TCRs with our composed list of unique and common SARS-CoV-2 epitopes (ref. to Methods section 2.4), we further classified it as SC2-unique.

Overall, our findings align with previously proposed mechanisms of effective T-cell response development, where timely (within two weeks) activation and expansion of T cells contribute to improved control of the virus (45, 49). Conversely, if the activation and/or expansion of the SARS-CoV-2 specific T-cells is delayed or dysfunctional, the virus multiplies unchecked, and the overactivated immune system causes more severe symptoms (7). Furthermore, we have observed the dominance of CD8+ T cells putatively recognizing SC2-unique rather CoV-common epitopes, which supports the previous report that in contrast to CD4+ T cells, most expanded CD8+ T cells did not cross-react with seasonal coronaviruses (27). Additionally, given the slight underrepresentation of SC2-unique epitopes compared to CoV-common in the trained TCRex models (19 SC2-unique vs 28 CoV-common), the prominence of SC2-unique TCRs in the progression of the T-cell response suggests that newly expanding T-cell clones may have a relatively high contribution to the SARS-CoV-2 immune response compared to potentially pre-existing cross-reactive T-cell clones.

Despite the congruence of our results, they should be interpreted with caution. Only a portion of critical and non-critical patients had the data available for both the first- and second-week post symptom onset, and not all individuals exhibited described trends within their respective groups. As such, half of the critical patients with continuous data (2 out of 4) demonstrated elevated CoV-common TCR frequency at week 2, which hints that even patients with the same disease severity do not have a uniform T-cell immune response to SARS-CoV-2. For instance, while pre-existing cross-reactive CD8+ T-cells may provide clinical protection to some individuals (22, 23), this protection has also been questioned (6, 26, 56). In agreement with the latter outlook (detrimental role of cross-reactive T cells), we have observed that disease outcome was fatal in three critical patients who had reached the highest frequencies of CoV-common TCRs during the first two weeks of COVID-19. In a recent study on metabolic changes in COVID-19 patients (55), metabolic pathways of several metabolically defined T-cell clusters positively correlated with disease severity. One cluster, comprising metabolically hyperactive, proliferative-exhausted SARS-CoV-2-specific T cells, enlarged with more severity COVID-19. Such dysfunctional state of expanded CoV-common T cells could explain their deleterious contribution noted here and in other studies.

The observed interpersonal variation in the CD8+ T-cell response within the same group in our dataset could also be attributed to the fact that current TCRex models do not cover the entire epitope space available to CD8+ T cells. Firstly, it has been experimentally evaluated that on average, one individual recognizes 17 MHC-I SARS-CoV-2 epitopes (57), which is more than was predicted with our recognition models for every patient. Secondly, TCRex models could be missing epitope-specific TCRs that are very different to the ones present in the training set. Lastly, although TCRex models are MHC-agnostic and thus could correctly predict epitope-TCR interaction for multiple TCR-epitope-MHC combinations (36), it has been demonstrated that some HLA-alleles strongly shape the set of recognized epitopes (18, 30, 58). Hence, the magnitude of CD8+ T-cell response is likely to be underestimated in our analysis at least for some of the patients. Therefore, the response could become more uniform across patients and TCR-specificity groups once there is enough experimental data, which are diverse in terms of CD8+ TCRs, epitopes and MHC-I, and recognition models are built for them.

Finally, there is evidence that multi-epitope T-cell response mitigates the effect of viral escape mutations (47). The broad and redundant response was valuable for SARS-CoV-2 control in our observations as well. Thus, monitoring the metrics of (specific) TCR repertoires and T-cell response in infected and vaccinated individuals could be helpful to better assess the necessity of an intervention to ensure and preserve adequate protection against any emerging SARS-CoV-2 variant. Moreover, since multiple studies have reported that reactivation of opportunistic herpesvirus (EBV, VZV, HHV-6, etc.) (59–61) and bystander activation of CMV-specific T cells (62) are associated with long COVID symptoms, resolving longitudinal dynamics of specific and cross-reactive T cells recognizing SARS-CoV-2 and aforementioned viruses could provide new insights into the development of post-acute COVID-19 sequelae. *In silico* approach can be leveraged to extract information such as specificity faster and with more flexibility than *in vitro* testing. Accordingly, our study offers a generalizable computational framework that complements the current standard of antibody-based assessment of COVID-19 immunity with TCR repertoire analysis.

## 5 Limitations of the study

The study acknowledges multiple limitations. Firstly, the analysis was constrained by a small sample size, which may affect the generalizability of the findings. Thus, they should be considered as preliminary indications, which warrant further research for verification. Next, our analysis may underestimate the magnitude of the CD8+ T-cell response in some patients due to limited coverage of the epitope space by current TCRex models. Furthermore, lack of information on the HLA profiles of the patients prevents a comprehensive examination of T-cell response, as true differences between the TCR repertoires of different patient groups may be concealed by HLA bias. Another limitation inherent to the analysis of TCR repertoires from blood samples is that the migration of T cells from the circulation to tissues remains hidden. Consequently, the observed intrapersonal dissimilarities in the prevalence of specific and cross-reactive TCRs could be reflective of the differences in the distribution rather than in the speed of generation of the respective T cells. Lastly, the epitopes classified here as unique to SARS-CoV-2 or common within coronaviruses, are expected to change over time. As variants evolve and more coronavirus species are discovered, epitopes may no longer be restricted to SARS-CoV-2. However, by considering the unique and shared epitopes that emerge with each variant’s set of mutations, a similar approach utilizing epitope-specific TCRs could help differentiate specific immune responses from broader immunity.

## Data availability statement

The datasets presented in this study can be found in online repositories. The names of the repository/repositories and accession number(s) can be found below: Files with TCR repertoires of each patient and files with specificity annotations of TCR beta sequences can be found in GitHub repository (https://github.com/apostovskaya/CovidTCRs/tree/main/data).

## Ethics statement

The studies involving human participants were reviewed and approved by Institutional Review Board, Institute of Tropical Medicine, Antwerp, Belgium and Ethics committee UZA, Antwerp University Hospital, Antwerp, Belgium. The patients/participants provided their written informed consent to participate in this study.

## Author contributions

PM, KV, KL, BO, and AP conceived and planned the experiments. AV, TdB, LvP, MvF, IB, EBo, CVD, CT, SHvI, EV, and EBa collected samples and generated the experimental data. AP, KM and PM developed the analytical framework and analyzed the data. AP, WA, GV, BO, KL, KV, and PM contributed to the interpretation of the results. AP took the lead in writing the manuscript. All authors contributed to the article and approved the submitted version.

## References

[1] Grifoni A Weiskopf D Ramirez SI Mateus J Dan JM Moderbacher CR. Targets of T cell responses to SARS-CoV-2 coronavirus in humans with COVID-19 disease and unexposed individuals. Cell (2020) 181:1489–1501.e15. doi: 10.1016/j.cell.2020.05.015 32473127 PMC7237901

[2] Gangaev A Ketelaars SLC Isaeva OI Patiwael S Dopler A Hoefakker K. Identification and characterization of a SARS-CoV-2 specific CD8+ T cell response with immunodominant features. Nat Commun (2021) 12:2593. doi: 10.1038/s41467-021-22811-y 33972535 PMC8110804

[3] Kared H Redd AD Bloch EM Bonny TS Sumatoh H Kairi F. SARS-CoV-2-specific CD8+ T cell responses in convalescent COVID-19 individuals. J Clin Invest (2021) 131:990–1000. doi: 10.1172/JCI145476 PMC7919723 33427749

[4] Wu F Liu M Wang A Lu L Wang Q Gu C. Evaluating the association of clinical characteristics with neutralizing antibody levels in patients who have recovered from mild COVID-19 in shanghai, China. JAMA Intern Med (2020) 180:1356–62. doi: 10.1001/JAMAINTERNMED.2020.4616 PMC9377417 32808970

[5] Swadling L Diniz MO Schmidt NM Amin OE Chandran A Shaw E. Pre-existing polymerase-specific T cells expand in abortive seronegative SARS-CoV-2. Nature (2022) 601:110–117. doi: 10.1038/s41586-021-04186-8 PMC8732273 34758478

[6] Chen Z John Wherry E. T Cell responses in patients with COVID-19. Nat Rev Immunol (2020) 20(9):529–36. doi: 10.1038/s41577-020-0402-6 PMC7389156 32728222

[7] Tan AT Linster M Tan CW le Bert N Chia WN Kunasegaran K. Early induction of functional SARS-CoV-2-specific T cells associates with rapid viral clearance and mild disease in COVID-19 patients. Cell Rep (2021) 34(6):108728. doi: 10.1016/j.celrep.2021.108728 33516277 PMC7826084

[8] Peng Y Mentzer AJ Liu G Yao X Yin Z Dong D. Broad and strong memory CD4+ and CD8+ T cells induced by SARS-CoV-2 in UK convalescent individuals following COVID-19. Nat Immunol (2020) 21:1336–45. doi: 10.1038/s41590-020-0782-6 PMC7611020 32887977

[9] Dan JM Mateus J Kato Y Hastie KM Yu ED Faliti CE. Immunological memory to SARS-CoV-2 assessed for up to 8 months after infection. Science (2021) 1979):371. doi: 10.1126/science.abf4063 PMC7919858 33408181

[10] Afkhami S D’Agostino MR Zhang A Stacey HD Marzok A Kang A. Respiratory mucosal delivery of next-generation COVID-19 vaccine provides robust protection against both ancestral and variant strains of SARS-CoV-2. Cell (2022) 85(5):896–915.e19. doi: 10.1016/j.cell.2022.02.005 PMC8825346 35180381

[11] Tarke A Coelho CH Zhang Z Dan JM Yu ED Methot N. SARS-CoV-2 vaccination induces immunological T cell memory able to cross-recognize variants from alpha to omicron. Cell (2022) 185:847–859.e11. doi: 10.1016/j.cell.2022.01.015 35139340 PMC8784649

[12] Yang LT Peng H Zhu ZL Li G Huang ZT Zhao ZX. Long-lived effector/central memory T-cell responses to severe acute respiratory syndrome coronavirus (SARS-CoV) s antigen in recovered SARS patients. Clin Immunol (2006) 120:171–8. doi: 10.1016/J.CLIM.2006.05.002 PMC7106132 16781892

[13] Liu WJ Zhao M Liu K Xu K Wong G Tan W. T-Cell immunity of SARS-CoV: implications for vaccine development against MERS-CoV. Antiviral Res (2017) 137:82–92. doi: 10.1016/J.ANTIVIRAL.2016.11.006 27840203 PMC7113894

[14] Oh HLJ Gan SKE Bertoletti A Tan YJ. Understanding the T cell immune response in SARS coronavirus infection. Emerg Microbes Infect (2019) 1(1):1–6. doi: 10.1038/emi.2012.26 PMC3636424 26038429

[15] le Bert N Tan AT Kunasegaran K Tham CYL Hafezi M Chia A. SARS-CoV-2-specific T cell immunity in cases of COVID-19 and SARS, and uninfected controls. Nature (2020) 584(7821):457–62. doi: 10.1038/s41586-020-2550-z 32668444

[16] Braun J Loyal L Frentsch M Wendisch D Georg P Kurth F. SARS-CoV-2-reactive T cells in healthy donors and patients with COVID-19. Nature (2020) 587(7833):, 270–274. doi: 10.1038/s41586-020-2598-9 32726801

[17] Mateus J Grifoni A Tarke A Sidney J Ramirez SI Dan JM. Selective and cross-reactive SARS-CoV-2 T cell epitopes in unexposed humans. Science (2020) 1979):370. doi: 10.1126/SCIENCE.ABD3871/SUPPL_FILE/PAPV2.PDF PMC7574914 32753554

[18] Lineburg KE Grant EJ Swaminathan S Chatzileontiadou DSM Szeto C Sloane H. CD8+ T cells specific for an immunodominant SARS-CoV-2 nucleocapsid epitope cross-react with selective seasonal coronaviruses. Immunity (2021) 54:1055–1065.e5. doi: 10.1016/J.IMMUNI.2021.04.006 33945786 PMC8043652

[19] Nesterenko PA McLaughlin J Tsai BL Burton Sojo G Cheng D Zhao D. HLA-A∗02:01 restricted T cell receptors against the highly conserved SARS-CoV-2 polymerase cross-react with human coronaviruses. Cell Rep (2021) 37(13):110167. doi: 10.1016/j.celrep.2021.110167 34919800 PMC8660260

[20] Loyal L Braun J Henze L Kruse B Dingeldey M Reimer U. Cross-reactive CD4+ T cells enhance SARS-CoV-2 immune responses upon infection and vaccination. Science (2021) 1979):374. doi: 10.1126/SCIENCE.ABH1823/SUPPL_FILE/SCIENCE.ABH1823_MDAR_REPRODUCIBILITY_CHECKLIST.PDF PMC10026850 34465633

[21] Kundu R Narean JS Wang L Fenn J Pillay T Fernandez ND. Cross-reactive memory T cells associate with protection against SARS-CoV-2 infection in COVID-19 contacts. Nat Commun (2022) 13:80. doi: 10.1038/s41467-021-27674-x 35013199 PMC8748880

[22] Mallajosyula V Ganjavi C Chakraborty S McSween AM Jimena Pavlovitch-Bedzyk A Wilhelmy J. CD8 + T cells specific for conserved coronavirus epitopes correlate with milder disease in patients with COVID-19. Sci Immunol (2021) 6:eabg5669. doi: 10.1126/sciimmunol.abg5669 34210785 PMC8975171

[23] Schulien I Kemming J Oberhardt V Wild K Seidel LM Killmer S. Characterization of pre-existing and induced SARS-CoV-2-specific CD8 + T cells. Nat Med (2021) 27:78–85. doi: 10.1038/S41591-020-01143-2 33184509

[24] Bacher P Rosati E Esser D Martini GR Saggau C Schiminsky E. Low-avidity CD4+ T cell responses to SARS-CoV-2 in unexposed individuals and humans with severe COVID-19. Immunity (2020) 53:1258–1271.e5. doi: 10.1016/j.immuni.2020.11.016 33296686 PMC7689350

[25] Dykema AG Zhang B Woldemeskel BA Garliss CC Cheung LS Choudhury D. Functional characterization of CD4+ T cell receptors crossreactive for SARS-CoV-2 and endemic coronaviruses. J Clin Invest (2021) 131(10):e146922. doi: 10.1172/JCI146922 33830946 PMC8121515

[26] Saggau C Martini GR Rosati E Meise S Messner B Kamps AK. The pre-exposure SARS-CoV-2-specific T cell repertoire determines the quality of the immune response to vaccination. Immunity (2022) 55(10):1924–1939.e5. doi: 10.1016/j.immuni.2022.08.003 PMC9372089 35985324

[27] Ferretti AP Kula T Wang Y Nguyen DMV Weinheimer A Dunlap GS. Unbiased screens show CD8+ T cells of COVID-19 patients recognize shared epitopes in SARS-CoV-2 that largely reside outside the spike protein. Immunity (2020) 53:1095–1107.e3. doi: 10.1016/j.immuni.2020.10.006 33128877 PMC7574860

[28] Schultheiß C Paschold L Simnica D Mohme M Willscher E von Wenserski L. Next-generation sequencing of T and b cell receptor repertoires from COVID-19 patients showed signatures associated with severity of disease. Immunity (2020) 53:442–455.e4. doi: 10.1016/J.IMMUNI.2020.06.024/ATTACHMENT/FC510DE8-66DD-4786-A2A4-31EF621363E8/MMC3.XLSX 32668194 PMC7324317

[29] Snyder TM Gittelman RM Klinger M May DH Osborne EJ Taniguchi R. Magnitude and dynamics of the T-cell response to SARS-CoV-2 infection at both individual and population levels. medRxiv (2020), 20165647. doi: 10.1101/2020.07.31.20165647 PMC11747429 39840037

[30] Wu D Kolesnikov A Yin R Guest JD Gowthaman R Shmelev A. Structural assessment of HLA-A2-restricted SARS-CoV-2 spike epitopes recognized by public and private T-cell receptors. Nat Commun (2022) 1(13):1–14. doi: 10.1038/s41467-021-27669-8 PMC8748687 35013235

[31] Shomuradova AS Vagida MS Sheetikov SA Zornikova KV Kiryukhin D Titov A. SARS-CoV-2 epitopes are recognized by a public and diverse repertoire of human T cell receptors. Immunity (2020) 53:1245–1257.e5. doi: 10.1016/j.immuni.2020.11.004 33326767 PMC7664363

[32] Minervina AA Komech EA Titov A Koraichi MB Rosati E Mamedov IZ. Longitudinal high-throughput tcr repertoire profiling reveals the dynamics of t-cell memory formation after mild covid-19 infection. Elife (2021) 10:1–17. doi: 10.7554/eLife.63502 PMC7806265 33399535

[33] Dash P Fiore-Gartland AJ Hertz T Wang GC Sharma S Souquette A. Quantifiable predictive features define epitope-specific T cell receptor repertoires. Nature (2017) 547:89–93. doi: 10.1038/nature22383 28636592 PMC5616171

[34] de Neuter N Bittremieux W Beirnaert C Cuypers B Mrzic A Moris P. On the feasibility of mining CD8+ T cell receptor patterns underlying immunogenic peptide recognition. Immunogenetics (2017) 70(3):159–68. doi: 10.1007/S00251-017-1023-5 28779185

[35] Glanville J Huang H Nau A Hatton O Wagar LE Rubelt F. Identifying specificity groups in the T cell receptor repertoire. Nature (2017) 547:94–8. doi: 10.1038/nature22976 PMC5794212 28636589

[36] Gielis S Moris P Bittremieux W de Neuter N Ogunjimi B Laukens K. Detection of enriched T cell epitope specificity in full T cell receptor sequence repertoires. Front Immunol (2019) 10:2820/BIBTEX. doi: 10.3389/FIMMU.2019.02820/BIBTEX 31849987 PMC6896208

[37] Meysman P de Neuter N Gielis S Bui Thi D Ogunjimi B Laukens K. On the viability of unsupervised T-cell receptor sequence clustering for epitope preference. Bioinformatics (2019) 35:1461–8. doi: 10.1093/BIOINFORMATICS/BTY821 30247624

[38] Meysman P Barton J Bravi B Cohen-Lavi L Karnaukhov V Lilleskov E. Benchmarking solutions to the T-cell receptor epitope prediction problem: IMMREP22 workshop report. ImmunoInformatics (2023) 9:100024. doi: 10.1016/J.IMMUNO.2023.100024

[39] World Health Organization. Guideline clinical management of COVID-19 patients: living guideline. (2021). Available at: https://www.who.int/publications/i/item/WHO-2019-nCoV-clinical-2021-2. WHO Reference Number: WHO/2019-nCoV/clinical/2021.2

[40] Corrie BD Marthandan N Zimonja B Jaglale J Zhou Y Barr E. iReceptor: a platform for querying and analyzing antibody/B-cell and T-cell receptor repertoire data across federated repositories. Immunol Rev (2018) 284:24–41. doi: 10.1111/IMR.12666 29944754 PMC6344122

[41] Bolotin DA Poslavsky S Mitrophanov I Shugay M Mamedov IZ Putintseva EV. MiXCR: software for comprehensive adaptive immunity profiling. Nat Methods (2015) 12(5):380–1. doi: 10.1038/nmeth.3364 25924071

[42] Zhou P Yang X Wang XG Hu B Zhang L Zhang W. A pneumonia outbreak associated with a new coronavirus of probable bat origin. Nature (2020) 579(7798):270–3. doi: 10.1038/s41586-020-2012-7 PMC7095418 32015507

[43] Altenhoff AM Glover NM Train CM Kaleb K Warwick Vesztrocy A Dylus D. The OMA orthology database in 2018: retrieving evolutionary relationships among all domains of life through richer web and programmatic interfaces. Nucleic Acids Res (2018) 46:D477. doi: 10.1093/NAR/GKX1019 29106550 PMC5753216

[44] Amicone M Borges V Alves MJ Isidro J Lıbia Z Duarte S. Mutation rate of SARS-CoV-2 and emergence of mutators during experimental evolution. Evol Med Public Health (2022) 10:142–55. doi: 10.1093/EMPH/EOAC010 PMC8996265 35419205

[45] Moss P. The T cell immune response against SARS-CoV-2. Nat Immunol (2022) 23(2):186–93. doi: 10.1038/s41590-021-01122-w 35105982

[46] Redd AD Nardin A Kared H Bloch EM Abel B Pekosz A. Minimal crossover between mutations associated with omicron variant of SARS-CoV-2 and CD8+ T-cell epitopes identified in COVID-19 convalescent individuals. mBio (2022) 13:e03617–21. doi: 10.1128/MBIO.03617-21/SUPPL_FILE/MBIO.03617-21-ST003.DOCX PMC8941890 35229637

[47] Redd AD Nardin A Kared H Bloch EM Pekosz A Laeyendecker O. CD8+ T-cell responses in COVID-19 convalescent individuals target conserved epitopes from multiple prominent SARS-CoV-2 circulating variants. Open Forum Infect Dis (2021) 8:ofab143. doi: 10.1093/OFID/OFAB143 34322559 PMC8083629

[48] Carter JA Preall JB Grigaityte K Goldfless SJ Jeffery E Briggs AW. Single T cell sequencing demonstrates the functional role of αβ TCR pairing in cell lineage and antigen specificity. Front Immunol (2019) 10:1516/BIBTEX. doi: 10.3389/FIMMU.2019.01516/BIBTEX 31417541 PMC6684766

[49] Sette A Crotty S. Adaptive immunity to SARS-CoV-2 and COVID-19. Cell (2021) 184(4):P861–880. doi: 10.1016/j.cell.2021.01.007 PMC7803150 33497610

[50] Su Y Chen D Yuan D Lausted C Choi J Dai CL. Multi-omics resolves a sharp disease-state shift between mild and moderate COVID-19. Cell (2020) 183:1479–1495.e20. doi: 10.1016/J.CELL.2020.10.037 33171100 PMC7598382

[51] Tan L Wang Q Zhang D Ding J Huang Q Tang YQ. Lymphopenia predicts disease severity of COVID-19: a descriptive and predictive study. Signal Transduction Targeted Ther (2020) 5(1):1–3. doi: 10.1038/s41392-020-0148-4 PMC7100419 32296069

[52] Elias G Meysman P Bartholomeus E de Neuter N Keersmaekers N Suls A. Preexisting memory CD4 T cells in naïve individuals confer robust immunity upon hepatitis b vaccination. Elife (2022) 11:e68388. doi: 10.7554/ELIFE.68388 35074048 PMC8824481

[53] Liu J Li S Liu J Liang B Wang X Wang H. Longitudinal characteristics of lymphocyte responses and cytokine profiles in the peripheral blood of SARS-CoV-2 infected patients. EBioMedicine (2020) 55:102763. doi: 10.1016/j.ebiom.2020.102763 32361250 PMC7165294

[54] André S Picard M Cezar R Roux-Dalvai F Alleaume-Butaux A Soundaramourty C. T Cell apoptosis characterizes severe covid-19 disease. Cell Death Differentiation (2022) 29(8):1486–99. doi: 10.1038/s41418-022-00936-x PMC8782710 35066575

[55] Lee JW Su Y Baloni P Chen D Pavlovitch-Bedzyk AJ Yuan D. Integrated analysis of plasma and single immune cells uncovers metabolic changes in individuals with COVID-19. Nat Biotechnol (2021) 40(1):110–20. doi: 10.1038/s41587-021-01020-4 PMC9206886 34489601

[56] Sette A Crotty S. Pre-existing immunity to SARS-CoV-2: the knowns and unknowns. Nat Rev Immunol (2020) 20:457–458. doi: 10.1038/s41577-020-0389-z PMC7339790 32636479

[57] Tarke A Sidney J Kidd CK Dan JM Ramirez SI Yu ED. Comprehensive analysis of T cell immunodominance and immunoprevalence of SARS-CoV-2 epitopes in COVID-19 cases. Cell Rep Med (2021) 2(2):100204. doi: 10.1016/j.xcrm.2021.100204 33521695 PMC7837622

[58] Francis JM Leistritz-Edwards D Dunn A Tarr C Lehman J Dempsey C. Allelic variation in class I HLA determines CD8+ T cell repertoire shape and cross-reactive memory responses to SARS-CoV-2. Sci Immunol (2021) 7:eabk3070. doi: 10.1126/sciimmunol.abk3070 PMC9017864 34793243

[59] Gold JE Okyay RA Licht WE Hurley DJ. Investigation of long COVID prevalence and its relationship to Epstein-Barr virus reactivation. Pathogens (2021) 10:763. doi: 10.3390/PATHOGENS10060763 34204243 PMC8233978

[60] Proal AD VanElzakker MB. Long COVID or post-acute sequelae of COVID-19 (PASC): an overview of biological factors that may contribute to persistent symptoms. Front Microbiol (2021) 12:698169/BIBTEX. doi: 10.3389/FMICB.2021.698169/BIBTEX 34248921 PMC8260991

[61] Davis HE McCorkell L Vogel JM Topol EJ. Long COVID: major findings, mechanisms and recommendations. Nat Rev Microbiol (2023) 21(3):133–46. doi: 10.1038/s41579-022-00846-2 PMC9839201 36639608

[62] Su Y Yuan D Chen DG Ng RH Wang K Choi J. Multiple early factors anticipate post-acute COVID-19 sequelae. Cell (2022) 185:881–895.e20. doi: 10.1016/J.CELL.2022.01.014 35216672 PMC8786632

