# Supplementary material for: Leveraging T-cell receptor – epitope recognition models to disentangle unique and cross-reactive T-cell response to SARS-CoV-2 during COVID-19 progression/resolution
Source: Front Immunol. 2023 May 31;14:1130876. doi: 10.3389/fimmu.2023.1130876 (PMC10264683; doi:10.3389/fimmu.2023.1130876)
Supplement: Supplementary file 1 [file DataSheet_1.docx]

Supplementary Material

Leveraging T‑cell receptor – epitope recognition models to disentangle unique and cross-reactive T‑cell response to SARS‑CoV‑2 during COVID-19 progression/resolution

Anna Postovskaya^1,2,3,4^, Alexandra Vujkovic^1,3,4^, Tessa de Block^4^, Lida van Petersen^5^, Maartje van Frankenhuijsen^5^, Isabel Brosius^5^, Emmanuel Bottieau^5^, Christophe Van Dijck^5,10^, Caroline Theunissen^5^, Sabrina H. van Ierssel^6^, Erika Vlieghe^6^, Esther Bartholomeus^3,7^, Kerry Mullan^1,2,3^, Wim Adriaensen^8^, Guido Vanham^9^, Benson Ogunjimi^3,10,11,12^, Kris Laukens^1,2,3^, Koen Vercauteren^4,†,*^, Pieter Meysman^1,2,3,†,*^

^1^Adrem Data Lab, Department of Computer Science, University of Antwerp, Antwerp, Belgium

^2^Biomedical Informatics Research Network Antwerp (BIOMINA), University of Antwerp, Antwerp, Belgium

^3^Antwerp Unit for Data Analysis and Computation in Immunology and Sequencing (AUDACIS), University of Antwerp, Antwerp, Belgium

^4^Clinical Virology Unit, Department of Clinical Sciences, Institute of Tropical Medicine, Antwerp, Belgium

^5^Department of Clinical Sciences, Institute of Tropical Medicine, Antwerp, Belgium

^6^Department of General Internal Medicine, Infectious Diseases and Tropical Medicine, Antwerp University Hospital, Edegem, Belgium

^7^Department of Medical Genetics, University of Antwerp, Antwerp, Belgium

^8^Clinical Immunology Unit, Department of Clinical Sciences, Institute of Tropical Medicine, Antwerp, Belgium

^9^Global Health Institute, University of Antwerp, Antwerp, Belgium

^10^Centre for Health Economics Research & Modeling Infectious Diseases (CHERMID), Vaccine & Infectious Disease Institute (VAXINFECTIO), University of Antwerp, Antwerp, Belgium

^11^Antwerp Center for Translational Immunology and Virology (ACTIV), Vaccine and Infectious Disease Institute, University of Antwerp, Antwerp, Belgium

^12^Department of Paediatrics, Antwerp University Hospital, Antwerp, Belgium

† These authors share last authorship

*** Correspondence:**Pieter Meysman
pieter.meysman@uantwerpen.be

Koen Vercauteren
kvercauteren@itg.be

# Supplementary Tables

Table S1 Summary of WHO criteria: COVID-19 disease severity in adults.

| Mild | Symptomatic COVID-19 with: | No clinical evidence of viral pneumonia* or hypoxia. |
| --- | --- | --- |
| Moderate | Clinical evidence of pneumonia* with: | RR ≤ 30 breaths/minute  AND  SpO2 ≥ 90% on room air |
| Severe |  | RR > 30 breaths/minute  OR  SpO2 < 90% on room air  OR  clinical signs of severe respiratory distress |
| Critical | ARDS with: | PaO2/FiO2 ≤ 300 mmHg  With PEEP or CPAP ≥ 5 cmH2O  OR  need for invasive ventilation |

*Clinical evidence of pneumonia: fever, cough, dyspnoea, fast breathing

Abbreviations: SpO2: oxygen saturation, PaO2/FiO2: arterial oxygen partial pressure (PaO2 in mmHg) to fractional inspired oxygen (FiO2), RR: Respiratory rate, ARDS: acute respiratory distress syndrome, PEEP: Positive end-expiratory pressure, CPAP: Continuous Positive Airway Pressure

Table S2 Patient and sample characteristics. The data is ordered by the COVID-19 severity and grouped by a patient. Time (day and week) refers to the time after the start of symptoms.

| **patient ID** | **COVID-19 status** | **COVID-19 severity** | **Patient group** | **day** | **week** | **T-cell population** | **total No. of TCRs** | **No. of unique TCRs** | **recognized SARS‑CoV‑2 epitopes** | **dataset** | **source** |
| --- | --- | --- | --- | --- | --- | --- | --- | --- | --- | --- | --- |
| **11** | recovered | mild | non-critical | 37 | 6 | cd8 | 50177 | 9741 | ['FLNGSCGSV', 'FVDGVPFVV', 'HTTDPSFLGRY', 'KLSYGIATV', 'VLWAHGFEL', 'YLQPRTFLL'] | "mixed" | Schultheiss et al. |
| **12** | recovered | mild | non-critical | 31 | 5 | cd8 | 45269 | 5974 | ['HTTDPSFLGRY', 'KLSYGIATV', 'MPASWVMRI'] | "mixed" | Schultheiss et al. |
| **13** | recovered | mild | non-critical | 36 | 6 | cd8 | 232656 | 21670 | ['FLNGSCGSV', 'HTTDPSFLGRY', 'ILHCANFNV', 'ITEEVGHTDLMAAY', 'MPASWVMRI', 'TPINLVRDL', 'VLWAHGFEL'] | "mixed" | Schultheiss et al. |
| **14** | recovered | mild | non-critical | 38 | 6 | cd8 | 224739 | 20562 | ['FLNGSCGSV', 'FLPRVFSAV', 'HLVDFQVTI', 'HTTDPSFLGRY', 'ILHCANFNV', 'NLDSKVGGNY', 'TPINLVRDL', 'YLNTLTLAV'] | "mixed" | Schultheiss et al. |
| **16** | recovered | mild | non-critical | 38 | 6 | cd8 | 251777 | 7334 | ['FLNGSCGSV'] | "mixed" | Schultheiss et al. |
| **17** | recovered | mild | non-critical | 39 | 6 | cd8 | 241379 | 17214 | ['FLNGSCGSV', 'HLVDFQVTI', 'ILHCANFNV', 'TLDSKTQSL', 'VLWAHGFEL', 'YLNTLTLAV'] | "mixed" | Schultheiss et al. |
| **18** | recovered | mild | non-critical | 39 | 6 | cd8 | 205546 | 9657 | ['FLNGSCGSV', 'FVDGVPFVV', 'HTTDPSFLGRY', 'ILHCANFNV', 'YLNTLTLAV'] | "mixed" | Schultheiss et al. |
| **26** | recovered | mild | non-critical | 45 | 7 | cd8 | 243824 | 18819 | ['FLPRVFSAV', 'HLVDFQVTI', 'HTTDPSFLGRY', 'ILHCANFNV', 'KPLEFGATSAAL', 'MPASWVMRI', 'TPINLVRDL'] | "mixed" | Schultheiss et al. |
| **27** | recovered | mild | non-critical | 45 | 7 | cd8 | 186983 | 15591 | ['ALSKGVHFV', 'FLNGSCGSV', 'FVDGVPFVV', 'HLVDFQVTI', 'ILHCANFNV', 'MPASWVMRI', 'NLDSKVGGNY', 'TPINLVRDL', 'YLQPRTFLL'] | "mixed" | Schultheiss et al. |
| **28** | recovered | mild | non-critical | 52 | 8 | cd8 | 207483 | 34579 | ['FLNGSCGSV', 'HLVDFQVTI', 'ILHCANFNV', 'MPASWVMRI', 'TPINLVRDL', 'YLDAYNMMI'] | "mixed" | Schultheiss et al. |
| **29** | recovered | mild | non-critical | 51 | 8 | cd8 | 180039 | 28651 | ['FLNGSCGSV', 'FLPRVFSAV', 'FPPTSFGPL', 'FVDGVPFVV', 'HLVDFQVTI', 'ILHCANFNV', 'TPINLVRDL', 'VLWAHGFEL', 'YLQPRTFLL'] | "mixed" | Schultheiss et al. |
| **32** | recovered | mild | non-critical | 34 | 5 | cd8 | 123000 | 19882 | ['FLNGSCGSV', 'HLVDFQVTI', 'ILHCANFNV', 'MPASWVMRI', 'TPINLVRDL'] | "mixed" | Schultheiss et al. |
| **33** | recovered | mild | non-critical | 39 | 6 | cd8 | 242458 | 28239 | ['FLNGSCGSV', 'FPPTSFGPL', 'ILHCANFNV', 'KLSYGIATV', 'MPASWVMRI', 'QECVRGTTVL', 'YLNTLTLAV'] | "mixed" | Schultheiss et al. |
| **34** | recovered | mild | non-critical | 39 | 6 | cd8 | 193187 | 17578 | ['FLNGSCGSV', 'FLPRVFSAV', 'ILHCANFNV', 'QECVRGTTVL', 'YFPLQSYGF'] | "mixed" | Schultheiss et al. |
| **35** | recovered | mild | non-critical | 47 | 7 | cd8 | 159895 | 12664 | ['FLNGSCGSV', 'FLPRVFSAV', 'FVDGVPFVV', 'HLVDFQVTI', 'HTTDPSFLGRY', 'VLWAHGFEL'] | "mixed" | Schultheiss et al. |
| **38** | recovered | mild | non-critical | 50 | 8 | cd8 | 147177 | 11653 | ['VLWAHGFEL'] | "mixed" | Schultheiss et al. |
| **6** | active | moderate | non-critical | 18 | 3 | cd8 | 103662 | 13701 | ['HTTDPSFLGRY', 'ILHCANFNV', 'NLDSKVGGNY', 'TPINLVRDL', 'VLWAHGFEL', 'YLQPRTFLL'] | "mixed" | Schultheiss et al. |
| **6** | recovered | moderate | non-critical | 23 | 4 | cd8 | 117083 | 12221 | ['FLNGSCGSV', 'HTTDPSFLGRY', 'NLDSKVGGNY', 'YLQPRTFLL'] | "mixed" | Schultheiss et al. |
| **7** | active | moderate | non-critical | 15 | 3 | cd8 | 107529 | 11181 | ['HLVDFQVTI', 'ILHCANFNV', 'VLWAHGFEL'] | "mixed" | Schultheiss et al. |
| **7** | active | moderate | non-critical | 20 | 3 | cd8 | 147800 | 23904 | ['EILDITPCSF', 'FLNGSCGSV', 'HLVDFQVTI', 'ILHCANFNV', 'KTSVDCTMYI', 'MPASWVMRI', 'TPINLVRDL', 'YLNTLTLAV'] | "mixed" | Schultheiss et al. |
| **7** | active | moderate | non-critical | 23 | 4 | cd8 | 81485 | 9284 | ['FLNGSCGSV', 'HLVDFQVTI', 'ILHCANFNV', 'KAYNVTQAF', 'MPASWVMRI', 'SSNVANYQK', 'YLNTLTLAV'] | "mixed" | Schultheiss et al. |
| **7** | recovered | moderate | non-critical | 27 | 4 | cd8 | 68120 | 9493 | ['FLNGSCGSV', 'KTSVDCTMYI', 'NLDSKVGGNY'] | "mixed" | Schultheiss et al. |
| **19** | active | moderate | non-critical | 9 | 2 | cd8 | 153813 | 10295 | ['EILDITPCSF', 'HTTDPSFLGRY', 'ILHCANFNV', 'NLDSKVGGNY', 'TPINLVRDL'] | "mixed" | Schultheiss et al. |
| **20** | active | moderate | non-critical | 24 | 4 | cd8 | 154706 | 9254 | ['NLDSKVGGNY', 'TPINLVRDL'] | "mixed" | Schultheiss et al. |
| **21** | active | moderate | non-critical | 20 | 3 | cd8 | 161562 | 6520 | ['FLPRVFSAV', 'HLVDFQVTI', 'HTTDPSFLGRY', 'ILHCANFNV', 'MPASWVMRI'] | "mixed" | Schultheiss et al. |
| **23** | active | moderate | non-critical | 23 | 4 | cd8 | 113730 | 696 | [''] | "mixed" | Schultheiss et al. |
| **24** | active | moderate | non-critical | 20 | 3 | cd8 | 225404 | 8016 | ['FLNGSCGSV', 'MPASWVMRI'] | "mixed" | Schultheiss et al. |
| **39** | active | moderate | non-critical | 1 | 1 | cd8 | 121953 | 6922 | ['FLNGSCGSV', 'FLPRVFSAV', 'TPINLVRDL'] | "mixed" | Schultheiss et al. |
| **imseq12** | active | moderate | non-critical | 9 | 2 | cd4 | 182948 | 14769 | ['FLNGSCGSV', 'FPPTSFGPL', 'HTTDPSFLGRY', 'KPLEFGATSAAL', 'YLQPRTFLL'] | "split" | in-house |
| **imseq12** | active | moderate | non-critical | 9 | 2 | cd8 | 223025 | 4815 | ['ALSKGVHFV', 'FLNGSCGSV', 'FLPRVFSAV', 'FPPTSFGPL', 'HTTDPSFLGRY', 'MPASWVMRI', 'VLWAHGFEL', 'YLQPRTFLL'] | "split" | in-house |
| **imseq12** | active | moderate | non-critical | 11 | 2 | cd4 | 560616 | 16487 | ['FLNGSCGSV', 'FPPTSFGPL', 'HTTDPSFLGRY', 'MPASWVMRI', 'NLDSKVGGNY'] | "split" | in-house |
| **imseq12** | active | moderate | non-critical | 11 | 2 | cd8 | 270648 | 6923 | ['ALSKGVHFV', 'FLNGSCGSV', 'FLPRVFSAV', 'FPPTSFGPL', 'HTTDPSFLGRY', 'YLQPRTFLL'] | "split" | in-house |
| **imseq13** | active | moderate | non-critical | 8 | 2 | cd8 | 396973 | 10791 | ['FLNGSCGSV', 'FPPTSFGPL', 'YFPLQSYGF'] | "split" | in-house |
| **imseq13** | active | moderate | non-critical | 11 | 2 | cd8 | 269797 | 7334 | ['FPPTSFGPL', 'HTTDPSFLGRY', 'ILHCANFNV', 'YFPLQSYGF'] | "split" | in-house |
| **imseq15** | active | moderate | non-critical | 14 | 2 | cd4 | 421515 | 17322 | ['FLNGSCGSV', 'HTTDPSFLGRY', 'YLQPRTFLL'] | "split" | in-house |
| **imseq15** | active | moderate | non-critical | 14 | 2 | cd8 | 218920 | 5441 | ['FLNGSCGSV', 'FPPTSFGPL', 'HTTDPSFLGRY', 'ILHCANFNV', 'KLSYGIATV', 'YLQPRTFLL'] | "split" | in-house |
| **imseq15** | active | moderate | non-critical | 20 | 3 | cd4 | 313305 | 14367 | ['HTTDPSFLGRY', 'ILHCANFNV'] | "split" | in-house |
| **imseq15** | active | moderate | non-critical | 20 | 3 | cd8 | 157961 | 4892 | ['FLNGSCGSV', 'FPPTSFGPL', 'HTTDPSFLGRY', 'ILHCANFNV'] | "split" | in-house |
| **imseq20** | active | moderate | non-critical | 16 | 3 | cd4 | 321554 | 6398 | [''] | "split" | in-house |
| **imseq20** | active | moderate | non-critical | 16 | 3 | cd8 | 215664 | 4181 | ['FLNGSCGSV', 'HLVDFQVTI'] | "split" | in-house |
| **imseq20** | active | moderate | non-critical | 19 | 3 | cd4 | 316147 | 17364 | ['EILDITPCSF', 'FLNGSCGSV', 'MPASWVMRI'] | "split" | in-house |
| **imseq20** | active | moderate | non-critical | 19 | 3 | cd8 | 307811 | 3654 | ['FLNGSCGSV'] | "split" | in-house |
| **imseq22** | active | moderate | non-critical | 16 | 3 | cd4 | 490683 | 16515 | ['FLNGSCGSV', 'HTTDPSFLGRY', 'ILHCANFNV', 'YLQPRTFLL'] | "split" | in-house |
| **imseq22** | active | moderate | non-critical | 16 | 3 | cd8 | 316833 | 7711 | ['FLNGSCGSV', 'FPPTSFGPL', 'HTTDPSFLGRY', 'RQLLFVVEV', 'TPINLVRDL', 'VLWAHGFEL', 'YLDAYNMMI', 'YLQPRTFLL'] | "split" | in-house |
| **imseq22** | active | moderate | non-critical | 23 | 4 | cd4 | 407646 | 9024 | ['FLNGSCGSV', 'MPASWVMRI'] | "split" | in-house |
| **imseq22** | active | moderate | non-critical | 23 | 4 | cd8 | 397411 | 10620 | ['EILDITPCSF', 'FLNGSCGSV', 'FPPTSFGPL', 'HTTDPSFLGRY', 'NLNESLIDL', 'RQLLFVVEV', 'TPINLVRDL', 'YLDAYNMMI', 'YLQPRTFLL'] | "split" | in-house |
| **imseq27** | active | moderate | non-critical | 7 | 1 | cd4 | 293974 | 7398 | ['FPPTSFGPL', 'YLQPRTFLL'] | "split" | in-house |
| **imseq27** | active | moderate | non-critical | 7 | 1 | cd8 | 283688 | 6394 | ['FADDLNQLTGY', 'FLNGSCGSV', 'FPPTSFGPL', 'HLVDFQVTI', 'HTTDPSFLGRY', 'KLPDDFTGCV', 'YLQPRTFLL'] | "split" | in-house |
| **imseq27** | active | moderate | non-critical | 14 | 2 | cd4 | 416178 | 19379 | ['FLNGSCGSV', 'FLPRVFSAV', 'HTTDPSFLGRY', 'KLSYGIATV', 'YLQPRTFLL'] | "split" | in-house |
| **imseq27** | active | moderate | non-critical | 14 | 2 | cd8 | 297148 | 5435 | ['FADDLNQLTGY', 'FLNGSCGSV', 'FPPTSFGPL', 'HLVDFQVTI', 'HTTDPSFLGRY', 'ILHCANFNV', 'YLQPRTFLL'] | "split" | in-house |
| **imseq5** | active | moderate | non-critical | 9 | 2 | cd4 | 245015 | 4527 | ['FLNGSCGSV', 'HTTDPSFLGRY', 'MPASWVMRI'] | "split" | in-house |
| **imseq5** | active | moderate | non-critical | 9 | 2 | cd8 | 421490 | 3309 | ['FLNGSCGSV', 'FPPTSFGPL', 'HTTDPSFLGRY', 'ILHCANFNV', 'MPASWVMRI'] | "split" | in-house |
| **imseq5** | active | moderate | non-critical | 11 | 2 | cd4 | 67267 | 839 | ['HTTDPSFLGRY'] | "split" | in-house |
| **imseq5** | active | moderate | non-critical | 11 | 2 | cd8 | 276002 | 7452 | ['FLNGSCGSV', 'FLPRVFSAV', 'FPPTSFGPL', 'HTTDPSFLGRY', 'ILHCANFNV', 'MPASWVMRI', 'NLDSKVGGNY', 'YLQPRTFLL'] | "split" | in-house |
| **imseq28** | active | severe | non-critical | 6 | 1 | cd4 | 395851 | 2535 | ['YLQPRTFLL'] | "split" | in-house |
| **imseq28** | active | severe | non-critical | 6 | 1 | cd8 | 185076 | 3001 | ['FPPTSFGPL', 'FVDGVPFVV'] | "split" | in-house |
| **imseq28** | active | severe | non-critical | 13 | 2 | cd4 | 267657 | 5694 | ['FLNGSCGSV', 'TPINLVRDL'] | "split" | in-house |
| **imseq28** | active | severe | non-critical | 13 | 2 | cd8 | 212354 | 3419 | ['FPPTSFGPL'] | "split" | in-house |
| **5** | active | critical | critical | 20 | 3 | cd8 | 100212 | 5468 | ['FVDGVPFVV', 'NLDSKVGGNY'] | "mixed" | Schultheiss et al. |
| **5** | active | critical | critical | 26 | 4 | cd8 | 126256 | 6186 | ['MPASWVMRI'] | "mixed" | Schultheiss et al. |
| **5** | active | critical | critical | 28 | 4 | cd8 | 72404 | 1680 | [''] | "mixed" | Schultheiss et al. |
| **5** | active | critical | critical | 32 | 5 | cd8 | 78614 | 4078 | ['FLNGSCGSV', 'ILHCANFNV', 'KLSYGIATV', 'MPASWVMRI', 'NLDSKVGGNY', 'VLWAHGFEL'] | "mixed" | Schultheiss et al. |
| **5** | active | critical | critical | 35 | 5 | cd8 | 177821 | 1431 | ['KTSVDCTMYI'] | "mixed" | Schultheiss et al. |
| **5** | active | critical | critical | 39 | 6 | cd8 | 151747 | 6506 | ['HLVDFQVTI', 'ILHCANFNV', 'MPASWVMRI', 'NLDSKVGGNY', 'TPINLVRDL'] | "mixed" | Schultheiss et al. |
| **5** | active | critical | critical | 41 | 6 | cd8 | 166547 | 8071 | ['NLDSKVGGNY', 'TPINLVRDL', 'YLQPRTFLL'] | "mixed" | Schultheiss et al. |
| **5** | active | critical | critical | 46 | 7 | cd8 | 196037 | 5509 | ['EILDITPCSF', 'FLPRVFSAV', 'TPINLVRDL'] | "mixed" | Schultheiss et al. |
| **10** | active | critical | critical | 25 | 4 | cd8 | 130525 | 7712 | ['HLVDFQVTI', 'HTTDPSFLGRY'] | "mixed" | Schultheiss et al. |
| **10** | active | critical | critical | 31 | 5 | cd8 | 152510 | 2927 | ['HLVDFQVTI', 'HTTDPSFLGRY'] | "mixed" | Schultheiss et al. |
| **10** | active | critical | critical | 33 | 5 | cd8 | 90871 | 1869 | ['HTTDPSFLGRY'] | "mixed" | Schultheiss et al. |
| **10** | active | critical | critical | 44 | 7 | cd8 | 149006 | 2594 | ['FPPTSFGPL'] | "mixed" | Schultheiss et al. |
| **10** | active | critical | critical | 46 | 7 | cd8 | 120122 | 2746 | ['HTTDPSFLGRY'] | "mixed" | Schultheiss et al. |
| **10** | active | critical | critical | 51 | 8 | cd8 | 184874 | 3202 | ['FLNGSCGSV', 'FPPTSFGPL', 'HLVDFQVTI', 'NLDSKVGGNY'] | "mixed" | Schultheiss et al. |
| **25** | active | critical | critical | 16 | 3 | cd8 | 270023 | 8452 | ['FLNGSCGSV', 'FPPTSFGPL', 'HLVDFQVTI', 'HTTDPSFLGRY', 'ILHCANFNV', 'YLQPRTFLL'] | "mixed" | Schultheiss et al. |
| **25** | active | critical | critical | 21 | 3 | cd8 | 182226 | 5418 | ['FLPRVFSAV'] | "mixed" | Schultheiss et al. |
| **40** | active | critical | critical | 13 | 2 | cd8 | 127651 | 2077 | [''] | "mixed" | Schultheiss et al. |
| **41** | active | critical | critical | 11 | 2 | cd8 | 106212 | 3490 | [''] | "mixed" | Schultheiss et al. |
| **44** | active | critical | critical | 15 | 3 | cd8 | 79980 | 6878 | ['FLNGSCGSV', 'FVDGVPFVV', 'NLDSKVGGNY', 'TLDSKTQSL'] | "mixed" | Schultheiss et al. |
| **imseq21** | active | critical | critical | 7 | 1 | cd4 | 242713 | 7046 | ['FLNGSCGSV', 'KEIDRLNEV'] | "split" | in-house |
| **imseq21** | active | critical | critical | 7 | 1 | cd8 | 268224 | 5446 | ['FLNGSCGSV', 'FPPTSFGPL', 'HTTDPSFLGRY', 'KLSYGIATV', 'MPASWVMRI', 'NLNESLIDL', 'YLQPRTFLL'] | "split" | in-house |
| **imseq21** | active | critical | critical | 13 | 2 | cd4 | 433177 | 11041 | ['FLNGSCGSV', 'KLPDDFTGCV'] | "split" | in-house |
| **imseq21** | active | critical | critical | 13 | 2 | cd8 | 335778 | 7132 | ['FPPTSFGPL', 'HTTDPSFLGRY', 'MPASWVMRI', 'YLQPRTFLL'] | "split" | in-house |
| **imseq21** | active | critical | critical | 21 | 3 | cd4 | 445044 | 18640 | ['FLPRVFSAV', 'HTTDPSFLGRY', 'MPASWVMRI', 'VLWAHGFEL', 'YLDAYNMMI'] | "split" | in-house |
| **imseq21** | active | critical | critical | 21 | 3 | cd8 | 325847 | 5973 | ['EILDITPCSF', 'FADDLNQLTGY', 'FLPRVFSAV', 'FPPTSFGPL', 'HTTDPSFLGRY', 'KLSYGIATV', 'MPASWVMRI', 'YLQPRTFLL'] | "split" | in-house |
| **imseq24** | active | critical | critical | 13 | 2 | cd4 | 318946 | 14754 | ['FLNGSCGSV', 'ILHCANFNV', 'YLQPRTFLL'] | "split" | in-house |
| **imseq24** | active | critical | critical | 13 | 2 | cd8 | 146463 | 946 | ['HTTDPSFLGRY'] | "split" | in-house |
| **imseq24** | active | critical | critical | 20 | 3 | cd4 | 272180 | 19798 | ['ILHCANFNV', 'YLQPRTFLL'] | "split" | in-house |
| **imseq24** | active | critical | critical | 26 | 4 | cd4 | 389850 | 14473 | ['FLNGSCGSV', 'HLVDFQVTI', 'HTTDPSFLGRY', 'YLQPRTFLL'] | "split" | in-house |
| **imseq24** | active | critical | critical | 26 | 4 | cd8 | 208251 | 2960 | ['FLNGSCGSV'] | "split" | in-house |
| **imseq25** | active | critical | critical | 5 | 1 | cd4 | 193326 | 10779 | [''] | "split" | in-house |
| **imseq25** | active | critical | critical | 5 | 1 | cd8 | 201760 | 3140 | ['FLNGSCGSV', 'FPPTSFGPL', 'HTTDPSFLGRY', 'ILHCANFNV', 'TPINLVRDL'] | "split" | in-house |
| **imseq25** | active | critical | critical | 12 | 2 | cd4 | 294805 | 12520 | ['MPASWVMRI'] | "split" | in-house |
| **imseq25** | active | critical | critical | 12 | 2 | cd8 | 181705 | 3895 | ['FPPTSFGPL', 'HTTDPSFLGRY', 'TPINLVRDL', 'YLQPRTFLL'] | "split" | in-house |
| **1** | active | fatal | critical | 2 | 1 | cd8 | 97424 | 1546 | ['FLNGSCGSV'] | "mixed" | Schultheiss et al. |
| **1** | active | fatal | critical | 6 | 1 | cd8 | 111024 | 1164 | [''] | "mixed" | Schultheiss et al. |
| **1** | active | fatal | critical | 8 | 2 | cd8 | 84277 | 2047 | ['MPASWVMRI'] | "mixed" | Schultheiss et al. |
| **1** | active | fatal | critical | 14 | 2 | cd8 | 111009 | 1953 | ['MPASWVMRI'] | "mixed" | Schultheiss et al. |
| **1** | active | fatal | critical | 23 | 4 | cd8 | 187602 | 445 | [''] | "mixed" | Schultheiss et al. |
| **1** | active | fatal | critical | 27 | 4 | cd8 | 195085 | 1174 | [''] | "mixed" | Schultheiss et al. |
| **2** | active | fatal | critical | 5 | 1 | cd8 | 111155 | 10804 | ['HLVDFQVTI', 'KPLEFGATSAAL', 'MPASWVMRI', 'VLWAHGFEL', 'YLNTLTLAV'] | "mixed" | Schultheiss et al. |
| **2** | active | fatal | critical | 9 | 2 | cd8 | 126924 | 5478 | ['FLNGSCGSV'] | "mixed" | Schultheiss et al. |
| **2** | active | fatal | critical | 11 | 2 | cd8 | 114373 | 4487 | ['ILHCANFNV', 'VLWAHGFEL'] | "mixed" | Schultheiss et al. |
| **3** | active | fatal | critical | 1 | 1 | cd8 | 97810 | 4126 | ['FLNGSCGSV', 'HTTDPSFLGRY'] | "mixed" | Schultheiss et al. |
| **3** | active | fatal | critical | 5 | 1 | cd8 | 107500 | 7135 | ['FLPRVFSAV', 'VLWAHGFEL', 'YLNTLTLAV'] | "mixed" | Schultheiss et al. |
| **8** | active | fatal | critical | 18 | 3 | cd8 | 115790 | 8391 | ['FLNGSCGSV', 'FLPRVFSAV', 'FVDGVPFVV', 'ILHCANFNV', 'TPINLVRDL'] | "mixed" | Schultheiss et al. |
| **8** | active | fatal | critical | 24 | 4 | cd8 | 106126 | 14181 | ['FLNGSCGSV', 'KTSVDCTMYI', 'NLDSKVGGNY'] | "mixed" | Schultheiss et al. |
| **9** | active | fatal | critical | 16 | 3 | cd8 | 136896 | 4416 | ['ILHCANFNV', 'KLSYGIATV', 'KTSVDCTMYI'] | "mixed" | Schultheiss et al. |
| **9** | active | fatal | critical | 22 | 4 | cd8 | 161978 | 5219 | ['FLNGSCGSV', 'FLPRVFSAV'] | "mixed" | Schultheiss et al. |

Table S3 SARS-CoV-2 epitope recognition model statistics. The data is grouped by protein and ordered by epitope uniqueness, i.e., in how many species, including SARS-CoV-2, an epitope is present (No. Species).

| Epitope | Uniqueness | No. Species | Protein | No. positive training TCRs | Balanced accuracy | AUC ROC | AUC PR |
| --- | --- | --- | --- | --- | --- | --- | --- |
| FADDLNQLTGY | SC2-unique | 1 | ORF1ab | 70 | 0.61 ± 0.04 | 0.7 ± 0.05 | 0.44 ± 0.11 |
| HTTDPSFLGRY | SC2-unique | 1 | ORF1ab | 5000 | 0.7 ± 0.01 | 0.9 ± 0.01 | 0.7 ± 0.02 |
| ITEEVGHTDLMAAY | SC2-unique | 1 | ORF1ab | 156 | 0.57 ± 0.02 | 0.78 ± 0.02 | 0.49 ± 0.04 |
| KPLEFGATSAAL | SC2-unique | 1 | ORF1ab | 344 | 0.59 ± 0.02 | 0.8 ± 0.04 | 0.49 ± 0.07 |
| SEISMDNSPNL | SC2-unique | 1 | ORF1ab | 95 | 0.52 ± 0.01 | 0.79 ± 0.05 | 0.45 ± 0.13 |
| SEVGPEHSLAEY | SC2-unique | 1 | ORF1ab | 250 | 0.54 ± 0.01 | 0.79 ± 0.01 | 0.42 ± 0.05 |
| TLIGDCATV | CoV-common | 9 | ORF1ab | 467 | 0.55 ± 0.02 | 0.73 ± 0.03 | 0.36 ± 0.04 |
| TLVPQEHYV | CoV-common | 11 | ORF1ab | 154 | 0.53 ± 0.03 | 0.72 ± 0.02 | 0.42 ± 0.07 |
| YLNTLTLAV | CoV-common | 12 | ORF1ab | 390 | 0.58 ± 0.02 | 0.8 ± 0.01 | 0.46 ± 0.04 |
| FPPTSFGPL | CoV-common | 13 | ORF1ab | 621 | 0.69 ± 0.01 | 0.85 ± 0.01 | 0.61 ± 0.01 |
| ILGLPTQTV | CoV-common | 13 | ORF1ab | 198 | 0.68 ± 0.02 | 0.83 ± 0.05 | 0.6 ± 0.08 |
| KLSYGIATV | CoV-common | 13 | ORF1ab | 2149 | 0.58 ± 0.0 | 0.84 ± 0.01 | 0.51 ± 0.01 |
| RQLLFVVEV | CoV-common | 13 | ORF1ab | 841 | 0.57 ± 0.01 | 0.83 ± 0.01 | 0.46 ± 0.02 |
| SSNVANYQK | CoV-common | 13 | ORF1ab | 74 | 0.61 ± 0.04 | 0.77 ± 0.04 | 0.45 ± 0.08 |
| VLWAHGFEL | CoV-common | 13 | ORF1ab | 695 | 0.65 ± 0.02 | 0.85 ± 0.02 | 0.6 ± 0.04 |
| YLDAYNMMI | CoV-common | 13 | ORF1ab | 197 | 0.62 ± 0.03 | 0.74 ± 0.03 | 0.42 ± 0.05 |
| KLWAQCVQL | CoV-common | 14 | ORF1ab | 266 | 0.55 ± 0.01 | 0.8 ± 0.02 | 0.46 ± 0.05 |
| MPASWVMRI | CoV-common | 15 | ORF1ab | 477 | 0.62 ± 0.02 | 0.82 ± 0.01 | 0.53 ± 0.01 |
| FLNRFTTTL | CoV-common | 16 | ORF1ab | 104 | 0.54 ± 0.03 | 0.72 ± 0.07 | 0.38 ± 0.12 |
| FLPRVFSAV | CoV-common | 16 | ORF1ab | 773 | 0.59 ± 0.02 | 0.84 ± 0.01 | 0.51 ± 0.03 |
| ILHCANFNV | CoV-common | 16 | ORF1ab | 185 | 0.61 ± 0.02 | 0.84 ± 0.03 | 0.52 ± 0.05 |
| FLNGSCGSV | CoV-common | 17 | ORF1ab | 2332 | 0.61 ± 0.0 | 0.86 ± 0.01 | 0.56 ± 0.02 |
| FVDGVPFVV | CoV-common | 27 | ORF1ab | 2420 | 0.54 ± 0.0 | 0.77 ± 0.01 | 0.37 ± 0.01 |
| IVDTVSALV | CoV-common | 27 | ORF1ab | 36 | 0.51 ± 0.03 | 0.76 ± 0.12 | 0.36 ± 0.18 |
| EILDITPCSF | SC2-unique | 1 | S (ORF2) | 68 | 0.63 ± 0.03 | 0.73 ± 0.06 | 0.46 ± 0.09 |
| FTISVTTEIL | SC2-unique | 1 | S (ORF2) | 159 | 0.55 ± 0.03 | 0.73 ± 0.03 | 0.37 ± 0.04 |
| LEPLVDLPI | SC2-unique | 1 | S (ORF2) | 367 | 0.54 ± 0.01 | 0.76 ± 0.02 | 0.35 ± 0.05 |
| LPPAYTNSF | SC2-unique | 1 | S (ORF2) | 127 | 0.53 ± 0.02 | 0.82 ± 0.03 | 0.45 ± 0.06 |
| NLDSKVGGNY | SC2-unique | 1 | S (ORF2) | 45 | 0.73 ± 0.06 | 0.96 ± 0.03 | 0.85 ± 0.05 |
| TLDSKTQSL | SC2-unique | 1 | S (ORF2) | 107 | 0.83 ± 0.06 | 0.97 ± 0.02 | 0.88 ± 0.06 |
| TPINLVRDL | SC2-unique | 1 | S (ORF2) | 249 | 0.64 ± 0.02 | 0.81 ± 0.03 | 0.53 ± 0.04 |
| YFPLQSYGF | SC2-unique | 1 | S (ORF2) | 357 | 0.53 ± 0.01 | 0.79 ± 0.03 | 0.4 ± 0.04 |
| YLQPRTFLL | SC2-unique | 1 | S (ORF2) | 315 | 0.91 ± 0.01 | 0.97 ± 0.01 | 0.92 ± 0.01 |
| NQKLIANQF | CoV-common | 2 | S (ORF2) | 51 | 0.76 ± 0.02 | 0.95 ± 0.02 | 0.78 ± 0.07 |
| KLPDDFTGCV | CoV-common | 6 | S (ORF2) | 1160 | 0.59 ± 0.01 | 0.84 ± 0.01 | 0.54 ± 0.02 |
| KTSVDCTMYI | CoV-common | 7 | S (ORF2) | 70 | 0.7 ± 0.05 | 0.89 ± 0.05 | 0.72 ± 0.08 |
| LLFNKVTLA | CoV-common | 8 | S (ORF2) | 39 | 0.51 ± 0.03 | 0.76 ± 0.04 | 0.37 ± 0.1 |
| NLNESLIDL | CoV-common | 11 | S (ORF2) | 132 | 0.59 ± 0.03 | 0.74 ± 0.02 | 0.42 ± 0.05 |
| KEIDRLNEV | CoV-common | 12 | S (ORF2) | 57 | 0.58 ± 0.02 | 0.7 ± 0.08 | 0.43 ± 0.1 |
| SEPVLKGVKL | CoV-common | 12 | S (ORF2) | 80 | 0.57 ± 0.02 | 0.79 ± 0.05 | 0.38 ± 0.1 |
| ALSKGVHFV | SC2-unique | 1 | ORF3a | 129 | 0.53 ± 0.01 | 0.79 ± 0.03 | 0.4 ± 0.09 |
| IPIQASLPF | SC2-unique | 1 | ORF3a | 104 | 0.51 ± 0.02 | 0.74 ± 0.09 | 0.35 ± 0.11 |
| WICLLQFAY | SC2-unique | 1 | M (ORF5) | 515 | 0.64 ± 0.02 | 0.81 ± 0.02 | 0.56 ± 0.06 |
| HLVDFQVTI | CoV-common | 8 | ORF6 | 69 | 0.61 ± 0.04 | 0.75 ± 0.06 | 0.44 ± 0.09 |
| QECVRGTTVL | CoV-common | 7 | ORF7a | 147 | 0.71 ± 0.02 | 0.85 ± 0.02 | 0.66 ± 0.04 |
| IQYIDIGNY | SC2-unique | 1 | ORF8 | 149 | 0.56 ± 0.03 | 0.82 ± 0.04 | 0.47 ± 0.06 |
| KAYNVTQAF | CoV-common | 2 | N (ORF9) | 708 | 0.61 ± 0.02 | 0.83 ± 0.01 | 0.57 ± 0.03 |

Table S4 Results of Mann-Whitney U test between the depth of SC2-unique and CoV-common TCR repertoires of critical and non-critical active patients at different weeks. P-values are corrected for multiple comparisons (Bonferroni). Significant p-values are in bold (alpha is 0.05).

| **week** | **adj_p-value critical** | **AUC critical** | **No. critical** | **adj_p-value non-critical** | **AUC  non-critical** | **No.  non-critical** |
| --- | --- | --- | --- | --- | --- | --- |
| **1** | **0.049** | 1 | 5 | 0.162 | 1 | 3 |
| **2** | 1 | 0.551 | 7 | 1 | 0.49 | 7 |
| **3** | 1 | 0.611 | 6 | 0.193 | 0.776 | 7 |
| **4** | 1 | 0.639 | 6 | 1 | 0.52 | 5 |
| **5** | 1 | 0.75 | 2 | NA | NA | NA |
| **6** | 1 | 0 | 1 | NA | NA | NA |
| **7** | 1 | 0.5 | 2 | NA | NA | NA |
| **8** | 1 | 0 | 1 | NA | NA | NA |

Table S5 Results of Mann-Whitney U test between critical and non-critical patients. P-values are corrected for multiple comparisons (Bonferroni). Significant p-values are in bold (alpha is 0.05).

| **week** | **parameter** | **adj_p-value** | **AUC** |
| --- | --- | --- | --- |
| **1** | total_TCRcount | 0.742 | 0.733 |
|  | %unique_TCRs | 1 | 0.533 |
|  | Freq_CoV-common_TCRs | 1 | 0.467 |
|  | Freq_SC2-unique_TCRs | 0.907 | 0.667 |
| **2** | N_CoV-common_Eps | **0.026** | 0.796 |
|  | normN_CoV-common_Eps | 0.054 | 0.816 |
|  | N_SC2-unique_Eps | 0.054 | 0.714 |
|  | normN_SC2-unique_Eps | 0.433 | 0.592 |

# Supplementary Figures


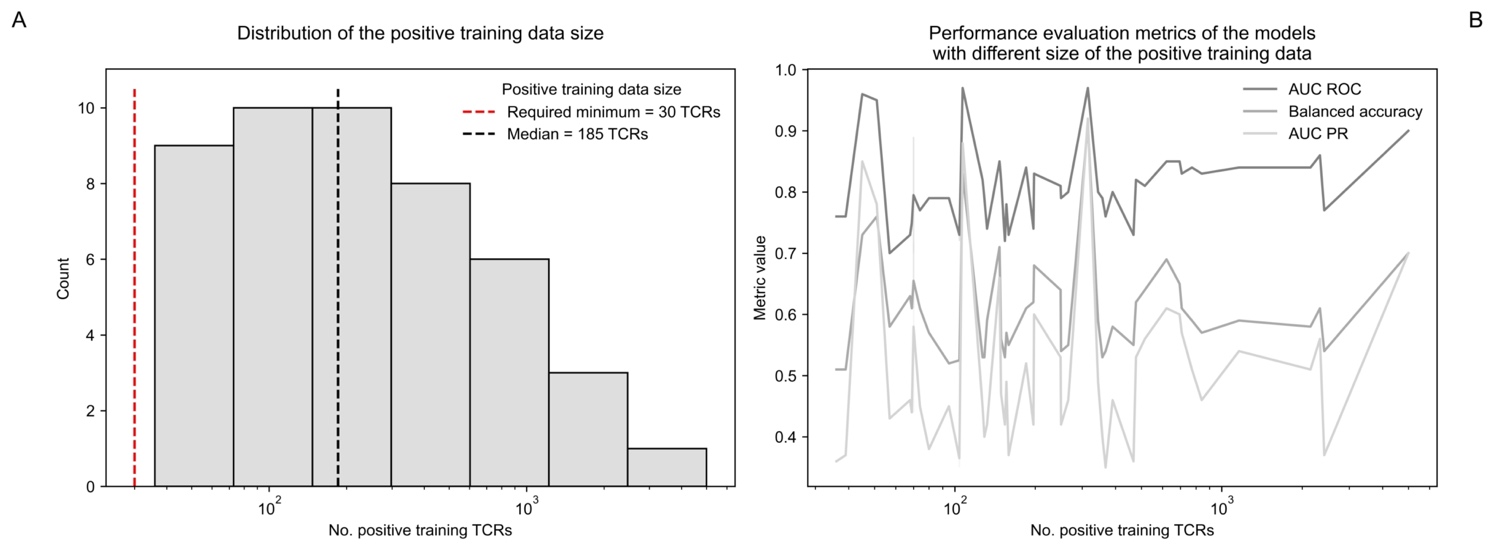


**Supplementary Figure 1.** (**A**) Distribution of the positive training data size (the number of unique TCRs experimentally validated to recognize a certain MHC-I epitope) among the trained TCRex models. All models had more than 30 unique TCRs in their positive training data as required by TCRex (median size was 185 TCRs). (**B**) Performance (AUC ROC, balanced accuracy, AUC PR) of the trained TCRex models against their positive training data size. The performance of all models was comparable, regardless of the size of the positive training data.


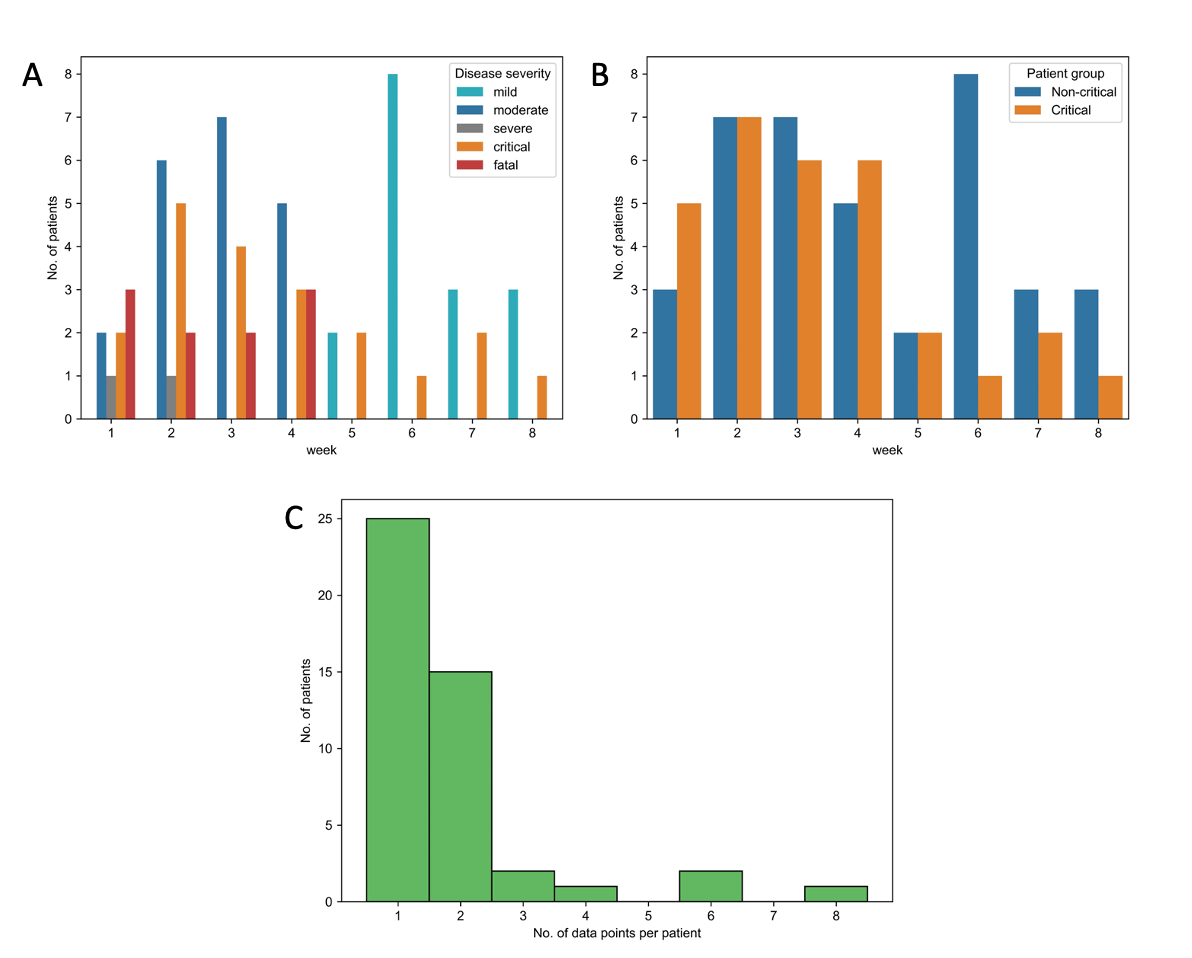


**Supplementary Figure 2.** Distribution of the available data points in the analyzed patient cohort (merged dataset) between individuals and weeks of the study. (**A**) The number of available patients at every week of the study had a high variation between different disease severity groups. (**B**) Once patients were divided into critical (critical, fatal) and non-critical (mild, moderate, severe) groups based on their disease severity, the number of available patients became comparable between those two groups at most weeks of the study. (**C**) Most of the patients in the merged dataset had 1-2 available data points.


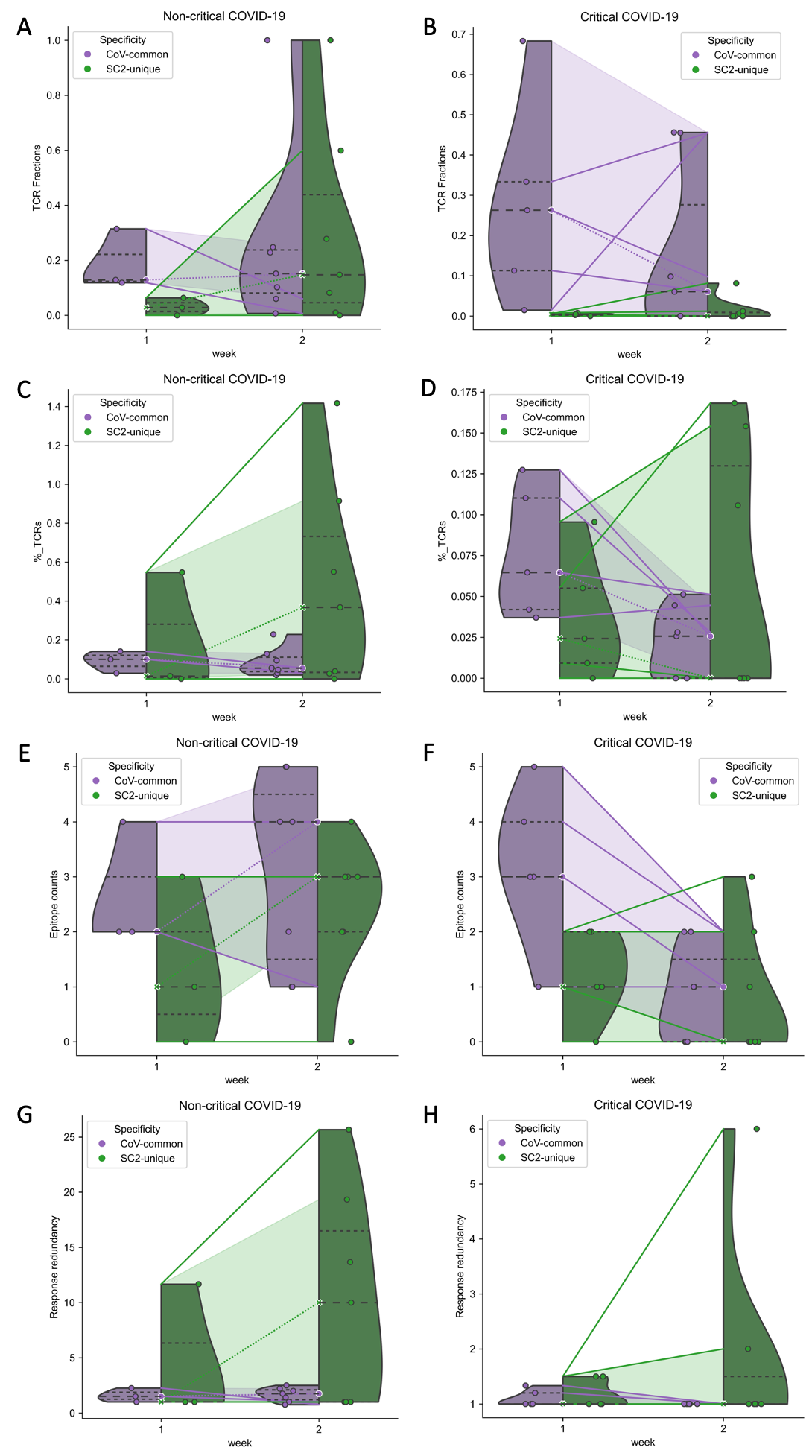


**Supplementary Figure 3.** The changes in the SC2-unique and CoV-common CD8+ TCR repertoires of critical and non-critical patients that occurred between the first and the second weeks of COVID-19: repertoire depth (**A**, **B**); repertoire breadth (**C**, **D**); response diversity **(E,** **F**); response redundancy (**G**, **H**). Dotted lines indicate a change in the median values (median data points are encircled with white); solid lines correspond to the dynamics of individual patients.


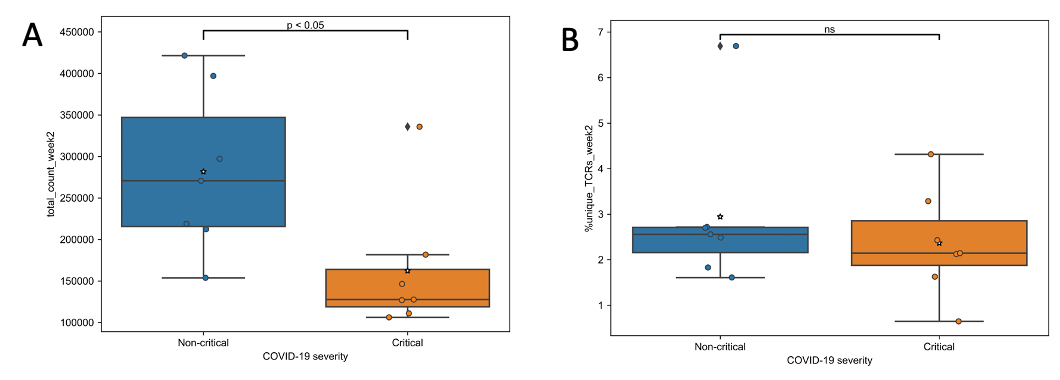


**Supplementary Figure 4.** During the second week of COVID-19, the total number of TCRs (**A**, Bonferroni corrected Mann–Whitney U test p=0.033) but not the percent of unique TCRs (**B**, Bonferroni corrected Mann–Whitney U test p=0.783) were significantly higher in non-critical compared to critical patients. Mean values are represented by a star.


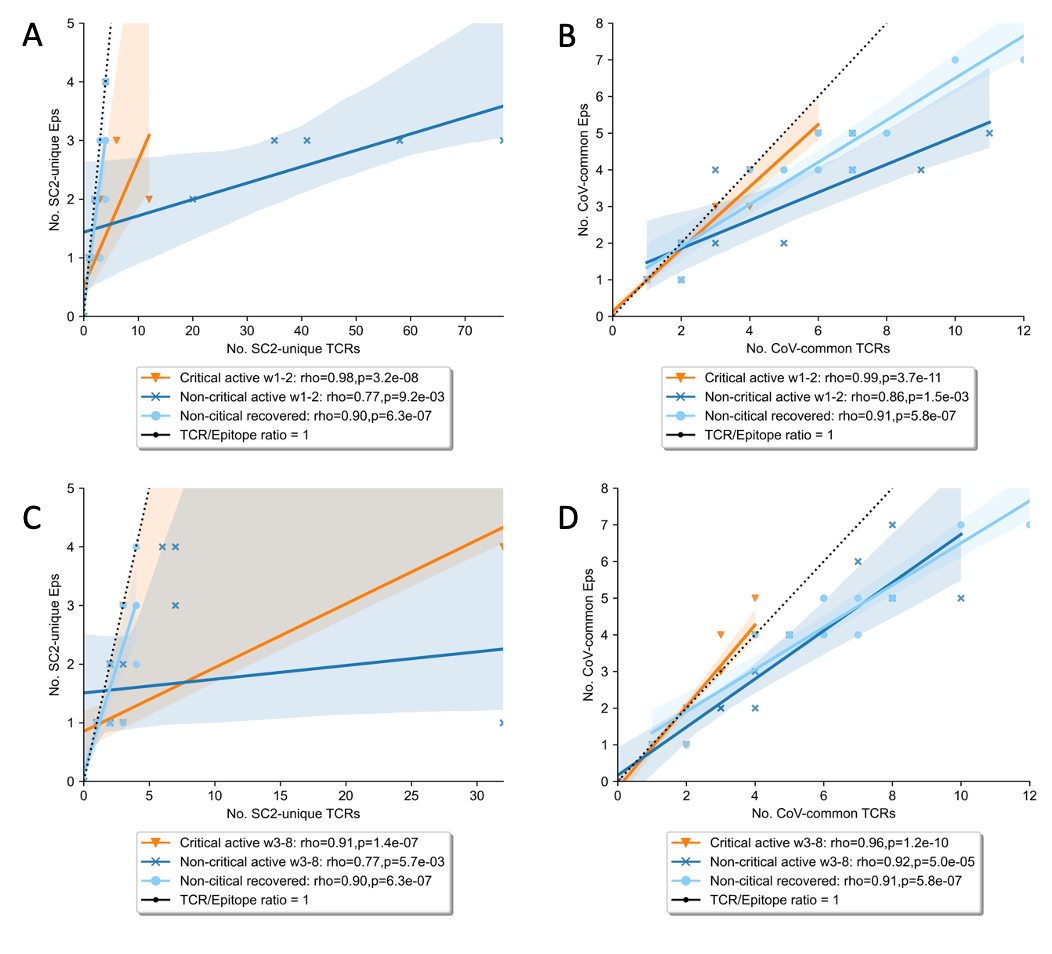


**Supplementary Figure 5.** The diversity and redundancy of the response during the initial stages of the disease (**A**, **B**: weeks 1-2 after symptom onset), late stages of the disease and recovery stage (**C**, **D**: weeks 3+ after symptom onset) differed between patients with critical and non-critical COVID­­-19.


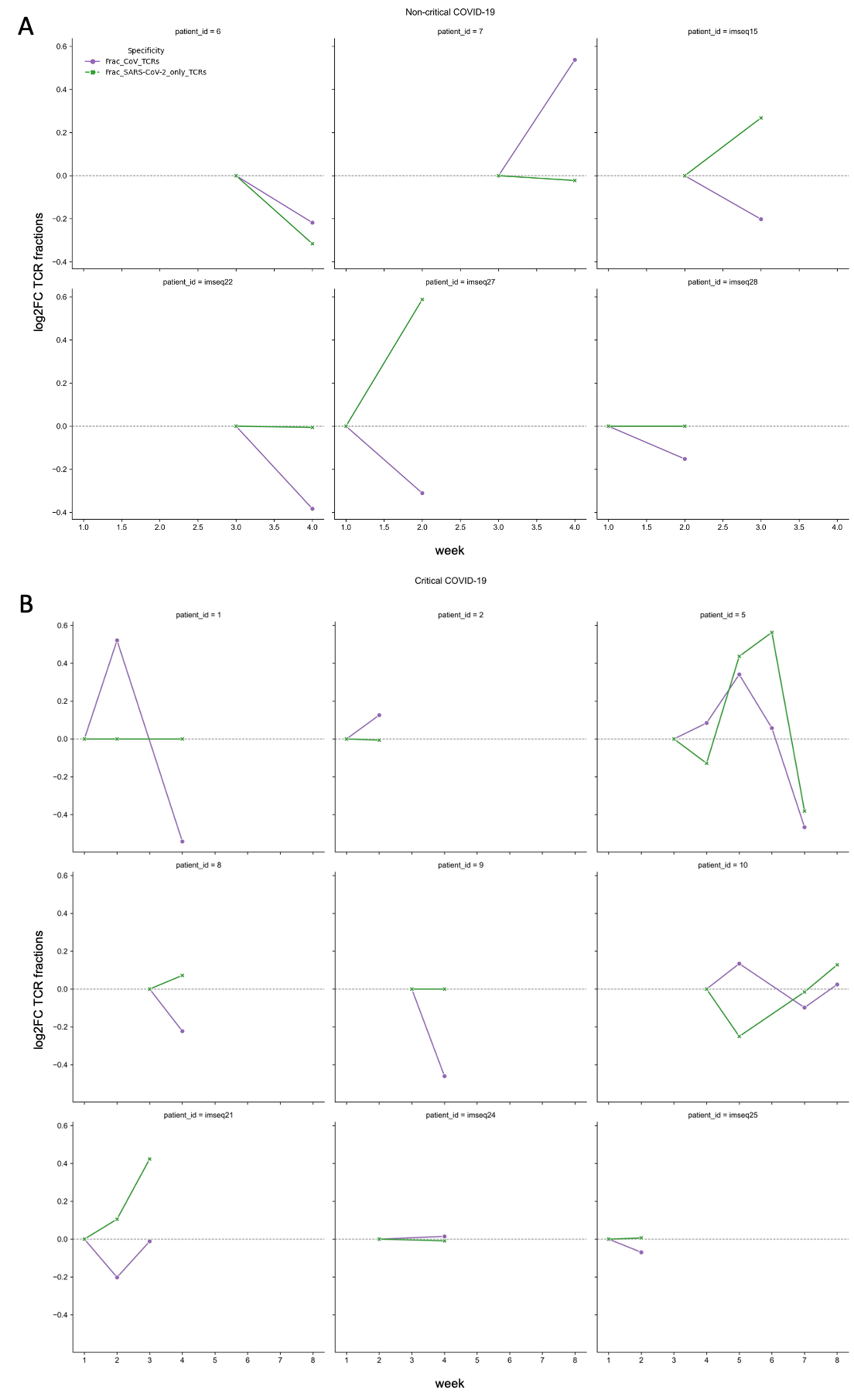


**Supplementary Figure 6.** The magnitude of the alterations in putative CoV-common (purple) and SC2-unique (green) TCR repertoires, expressed as log2 fold changes in the relative frequencies of TCRs (depth of the repertoire) in non-critical **(A)** and critical **(B)** COVID-19 patients. Data points of the same patient are connected with lines to visualize temporal dynamics.


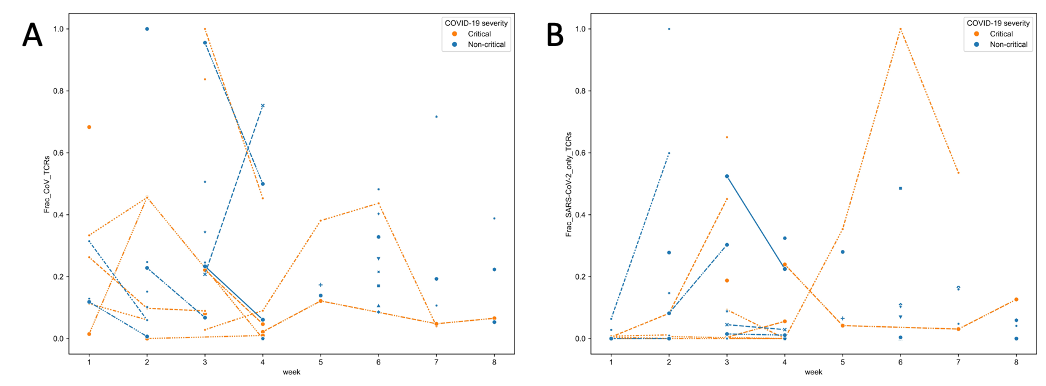


**Supplementary Figure 7.** The evolution of putative CoV-common (**A**) and SC2-unique (**B**) TCR repertoires over time in individual critical (orange) and non-critical (blue) patients, expressed as changes in the relative frequencies of TCRs (depth of the repertoire). Raw data points of the same patient are connected with lines to visualize longitudinal dynamics.


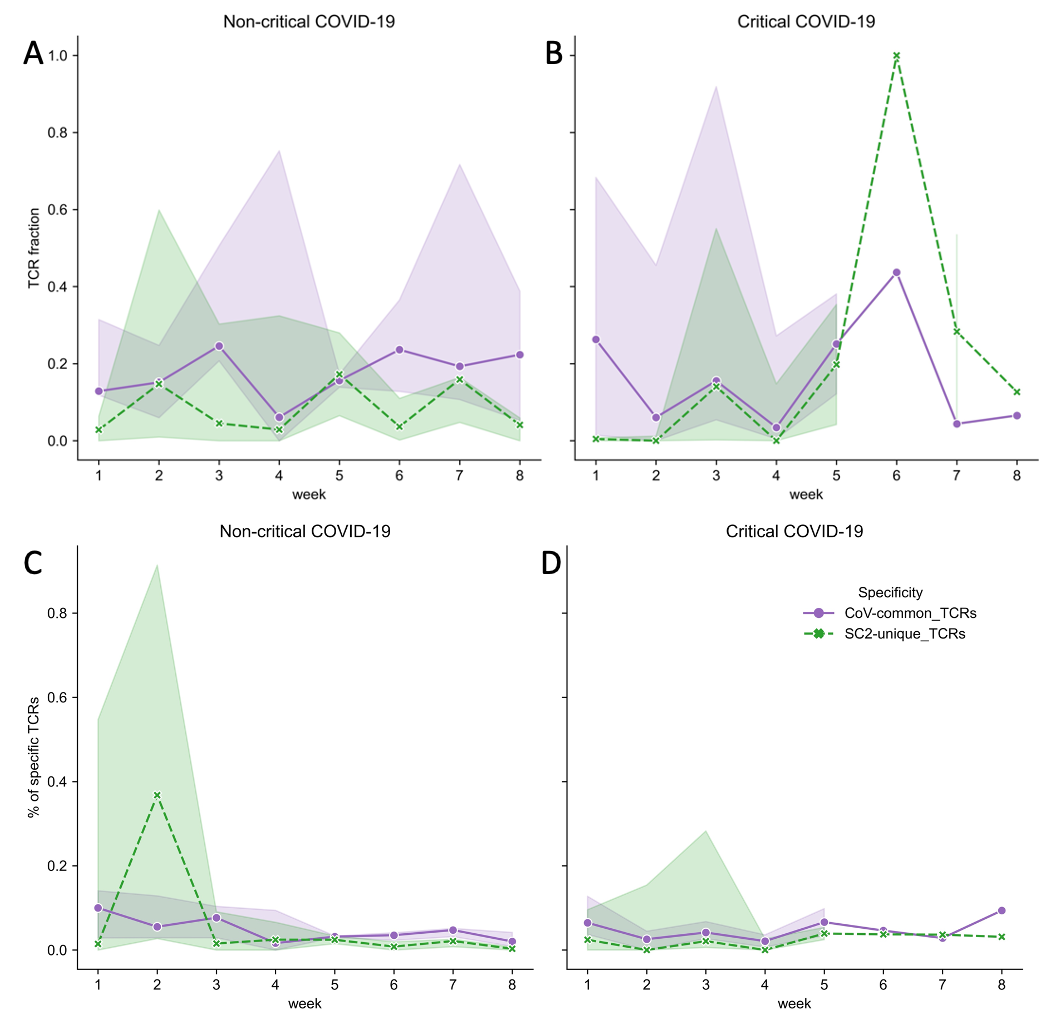


**Supplementary Figure 8.** The dynamics of putative SARS-CoV-2 T cells reactive to epitopes unique to SARS-CoV-2 (SC2-unique, green) or shared with other species of the Nidovirales order (CoV-common, purple) expressed as repertoire depth **(A,** **B**) and breadth **(C,** **D**). Lines represent an estimate of the central tendency of the respective values combined within each disease severity group with a 95% confidence interval shown as shadow areas when multiple data points are available at overlapping time points.

# Supplementary Material 1

Assembled single-cell CD8+ TCR sequences generated by Su et al. (Su et al., 2020) were downloaded from Array Express (accession number E-MTAB-9357); and only TCRs with paired and functional alpha and beta chains were kept. In case multiple beta chains were available for one cell, only the first sequence was used in the further analysis. To allow the comparison of the results with the assembled “merged” dataset, the disease severity annotations of this single-cell dataset have been converted. As such, patients with the grade of at least 6 on the WHO ordinal scale and all patients admitted to an intensive care unit (ICU) were considered to be critically ill (median age 65) and others - non-critically ill (median age 63.5). If an individual had different COVID-19 severity at different time points, the highest degree of severity was retained for all time points resulting in 1 disease severity annotation per patient. From the available blood samples, only those that were collected within the first week after the onset of symptoms or diagnosis were included in the analysis as the immune response can evolve drastically during the disease progression and make results incomparable between the datasets.

Specificity of patients’ TCR beta sequences was determined using TCRex recognition models as described in the respective methods section. To identify more SARS-CoV-2-specific TCRs, TCR beta sequences of all patients were clustered based on CDR3 sequence similarity using ClusTCR v.1.0.2 (Valkiers et al., 2021) with default parameters. Within one cluster, all TCRs were considered to have the same epitope specificity. Thus, if a cluster contained TCRs, for which specificity had been identified with TCRex, all TCRs within this cluster were annotated with this specificity. 221 and 348 specific TCRs were identified in samples from patients with critical (n=20) and non-critical (n=30) COVID-19 presentation, respectively. Next, TCR repertoire metrics were calculated for each patient as described in the respective methods section. The depths of SC2-unique and CoV-common TCR repertoires during week 1 after the symptom onset were compared between critical and non-critical patient groups.

In agreement with the findings in the “merged” dataset (Fig.3), both patients with critical (Bonferroni corrected MU p=0.003, AUC=0.77) and non-critical (Bonferroni corrected MU p=1.2e-06, AUC=0.86) COVID-19 severity, had higher prevalence of TCRs specific to CoV-common than SC2-unique epitopes (Fig.S9). Furthermore, no significant difference in the total number of TCRs (MU p=0.097), the percent of unique TCRs (MU p=0.364) and the depth of putative CoV-common (MU p=0.328) and SC2-unique (MU p=0.084) TCR repertoires was detected between two patient groups at this time.


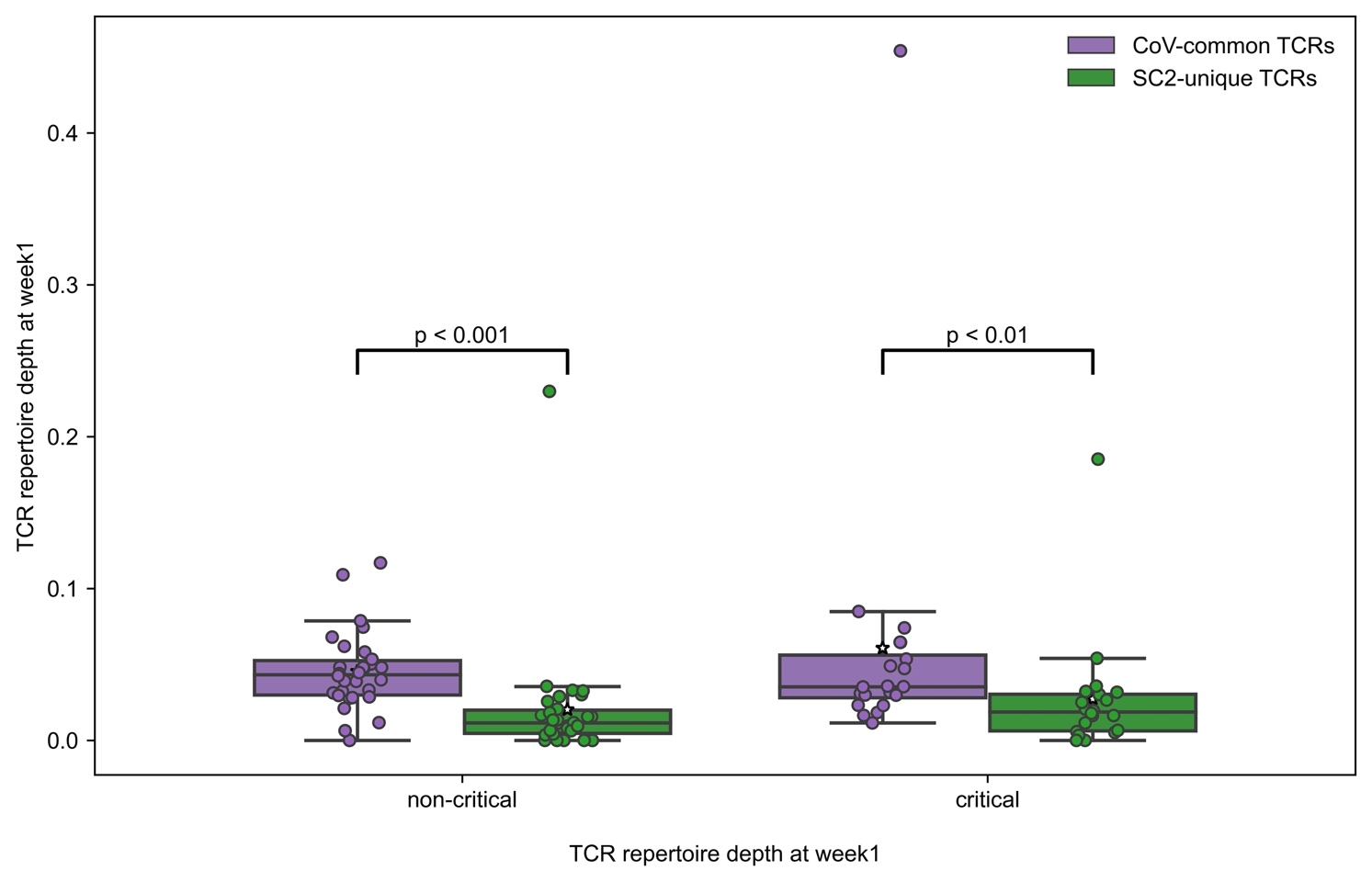


**Supplementary Figure 9** During the first week of COVID-19, relative frequencies of TCRs (depth of the repertoire) predicted to recognize CoV-common epitopes were significantly higher than of putative SC2-unique TCRs in all patients from the single-cell cohort, with non-critical (Bonferroni corrected Mann–Whitney U test p=1.2e-06, AUC=0.86, n=30) and critical (Bonferroni corrected Mann–Whitney U test p=0.003, AUC=0.77, n=20) COVID-19. Mean values are represented by a white star.

For closer examination of CD8+ T cell populations, we selected all patients (n=5) who had higher abundance of SC2-unique than CoV-common TCRs during supposed week 1 of the disease. For 4 out of those 5 patients, only the first day of COVID-19 severity assessment but not the exact date of the symptom onset was available. Therefore, it is likely that they were already at a later stage of the disease, thus allowing a glimpse into further development of the CD8+ T-cell response.

First, clonal expansion of all CD8+ T cells of those 4 patients (2 from each patient group: INCOV012, INCOV145 (non-critical COVID-19) and INCOV106, INCOV053 (critical COVID-19)) was investigated. Next, the phenotypes of 3 groups of T cells were identified for 2 patients (non-critical INCOV012 and critical INCOV106) who were representative of their respective disease severity groups in terms of diversity of SARS-CoV-2-specific TCRs and for whom more than 1 TCR was annotated as SC2-unique and CoV-common. To this end, the gene expression and TCR sequencing data of selected individuals were processed using our in-house program STEGO.R (R version 4.2.3). Briefly, STEGO converted the array data to the Seurat input format. The expression data was filtered for poor quality cells following the standard Seurat v.4.3.0 pipeline (Butler et al., 2018). Filtered and annotated with specificity (SC2-unique, CoV-common, Unknown) TCR sequencing data were added to the Seurat meta-data. The files were merged using Harmony v.0.1.1 (Korsunsky et al., 2019) and functionally annotated using scGATE v.1.4.1 (Andreatta et al., 2022) with custom cell type markers from Su et al. (Su et al., 2020).

We observed that only CD8+ T cells of patients with non-critical COVID-19 (INCOV012 and INCOV145) underwent clonal expansion to a large clone size (Fig.S10A, 2 left panels). The bulk of CD8+ T-cell populations of critical COVID-19 patients (INCOV106 and INCOV053) constituted singleton T cells (Fig.S10A, 2 right panels). T cells with SC2-unique TCRs, with CoV-common TCRs, and with TCRs that formed into clusters but could not have been previously annotated with TCRex (Unknown specificity) displayed different phenotypes in 1 non-critical (INCOV012) and 1 critical (INCOV106) COVID-19 patient. The predominant part of all SC2-unique T cells had effector-like phenotype with expression of *GNLY*, *PFR1* and *GZMB* (Fig.S10B). T cells with CoV-common TCRs, represented as single or small clones in both patients, had a mix of effector, memory and naïve phenotypes; naïve and effector T cells had the biggest share in critical INCOV106 and non-critical INCOV012, respectively (Fig.S10B). Notably, among the T cells with unknown specificity, some were of memory phenotype only in non-critical INCOV012 (Fig.S10B, left panel) whereas critical INCOV106 had a greater fraction of naïve T cells (Fig.S10B, right panel). Greater prevalence of naïve CD8+ T cells in critical patient and memory CD8+ T cells in non-critical patient as well as the dominance of effector CD8+ T cells putatively recognizing SC2-unique epitopes supports and enhances our earlier findings from the bulk TCR data (“merged” dataset).


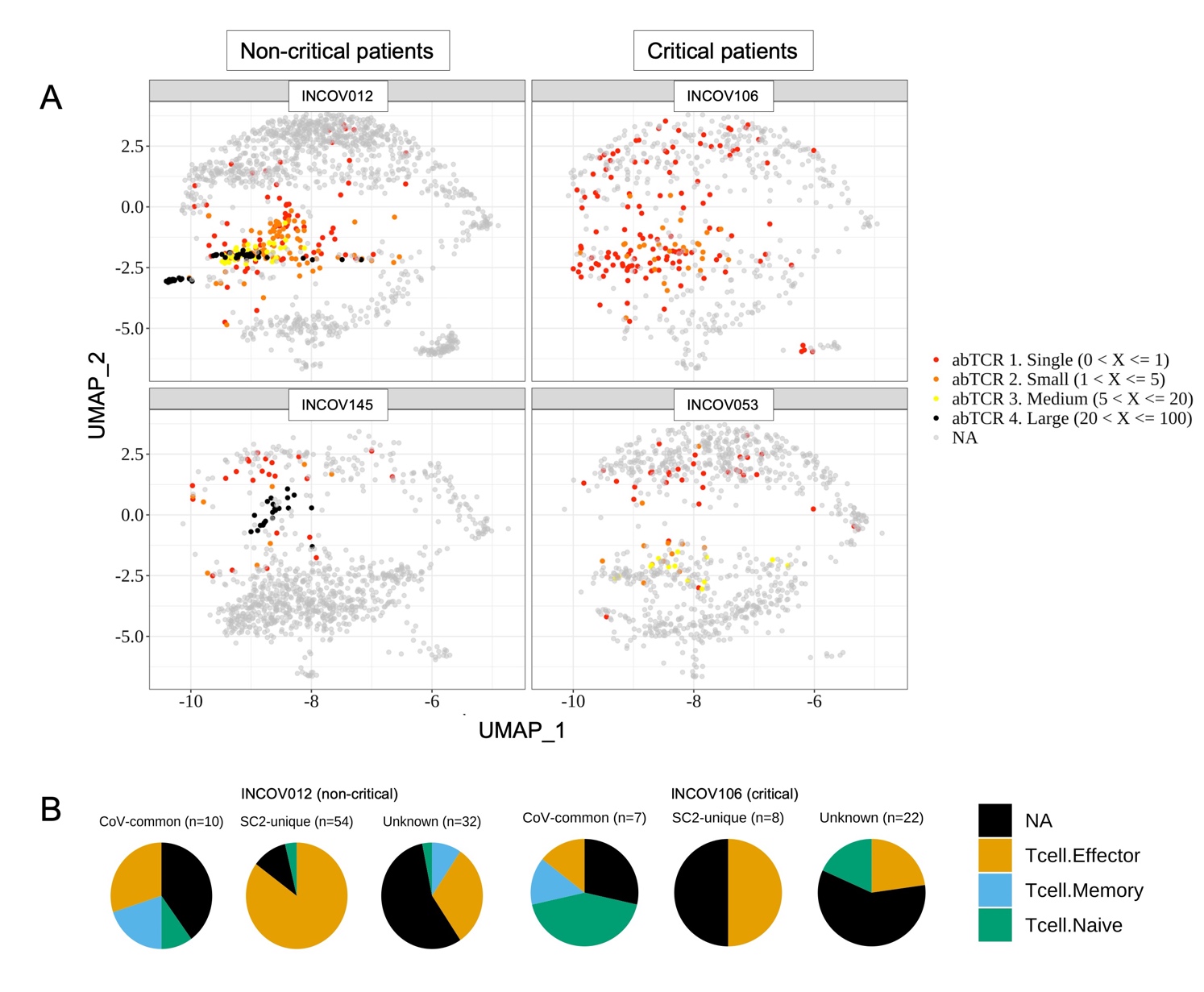


**Supplementary Figure 10** Clonal and functional information about CD8+ T cells of patients with non-critical and critical COVID-19 who already had more developed SC2-unique T-cell response. (**A**) UMAP embedding showcases the size distribution of all CD8+ T-cell clones for 4 patients from each disease severity group. (**B**) Pie chart depicts the phenotypes of T cells with SC2-unique TCRs, CoV-common TCRs and TCRs that formed into clusters but could not be annotated with TCRex (Unknown) for one patient from each disease severity group. NA = annotation is not available.

References

Andreatta, M., Berenstein, A.J., Carmona, S.J., 2022. scGate: marker-based purification of cell types from heterogeneous single-cell RNA-seq datasets. Bioinformatics 38, 2642–2644. https://doi.org/10.1093/BIOINFORMATICS/BTAC141

Butler, A., Hoffman, P., Smibert, P., Papalexi, E., Satija, R., 2018. Integrating single-cell transcriptomic data across different conditions, technologies, and species. Nat Biotechnol 36, 411. https://doi.org/10.1038/NBT.4096

Korsunsky, I., Millard, N., Fan, J., Slowikowski, K., Zhang, F., Wei, K., Baglaenko, Y., Brenner, M., Loh, P. ru, Raychaudhuri, S., 2019. Fast, sensitive, and accurate integration of single cell data with Harmony. Nat Methods 16, 1289. https://doi.org/10.1038/S41592-019-0619-0

Su, Y., Chen, D., Yuan, D., Lausted, C., Choi, J., Dai, C.L., Voillet, V., Duvvuri, V.R., Scherler, K., Troisch, P., Baloni, P., Qin, G., Smith, B., Kornilov, S.A., Rostomily, C., Xu, A., Li, J., Dong, S., Rothchild, A., Zhou, J., Murray, K., Edmark, R., Hong, S., Heath, J.E., Earls, J., Zhang, R., Xie, J., Li, S., Roper, R., Jones, L., Zhou, Y., Rowen, L., Liu, R., Mackay, S., O’Mahony, D.S., Dale, C.R., Wallick, J.A., Algren, H.A., Zager, M.A., Wei, W., Price, N.D., Huang, S., Subramanian, N., Wang, K., Magis, A.T., Hadlock, J.J., Hood, L., Aderem, A., Bluestone, J.A., Lanier, L.L., Greenberg, P.D., Gottardo, R., Davis, M.M., Goldman, J.D., Heath, J.R., 2020. Multi-Omics Resolves a Sharp Disease-State Shift between Mild and Moderate COVID-19. Cell 183, 1479-1495.e20. https://doi.org/10.1016/J.CELL.2020.10.037

Valkiers, S., Van Houcke, M., Laukens, K., Meysman, P., 2021. ClusTCR: a python interface for rapid clustering of large sets of CDR3 sequences with unknown antigen specificity. Bioinformatics 37, 4865–4867. https://doi.org/10.1093/BIOINFORMATICS/BTAB446
